# Computational Multiscale Solvers for Continuum Approaches

**DOI:** 10.3390/ma12050691

**Published:** 2019-02-26

**Authors:** Francisco Montero-Chacón, José A. Sanz-Herrera, Manuel Doblaré

**Affiliations:** 1 Departamento de Ingeniería, Universidad Loyola Andalucía, 41014 Seville, Spain; 2 Escuela Técnica Superior de Ingeniería, Universidad de Sevilla, 41092 Seville, Spain; jsanz@us.es; 3 Aragon Institute of Engineering Research (I3A), University of Zaragoza, 50018 Zaragoza, Spain; mdoblare@unizar.es

**Keywords:** multiscale analysis, homogenization, proper generalized decomposition, computational simulation

## Abstract

Computational multiscale analyses are currently ubiquitous in science and technology. Different problems of interest—e.g., mechanical, fluid, thermal, or electromagnetic—involving a domain with two or more clearly distinguished spatial or temporal scales, are candidates to be solved by using this technique. Moreover, the predictable capability and potential of multiscale analysis may result in an interesting tool for the development of new concept materials, with desired macroscopic or apparent properties through the design of their microstructure, which is now even more possible with the combination of nanotechnology and additive manufacturing. Indeed, the information in terms of field variables at a finer scale is available by solving its associated localization problem. In this work, a review on the algorithmic treatment of multiscale analyses of several problems with a technological interest is presented. The paper collects both classical and modern techniques of multiscale simulation such as those based on the proper generalized decomposition (PGD) approach. Moreover, an overview of available software for the implementation of such numerical schemes is also carried out. The availability and usefulness of this technique in the design of complex microstructural systems are highlighted along the text. In this review, the fine, and hence the coarse scale, are associated with continuum variables so atomistic approaches and coarse-graining transfer techniques are out of the scope of this paper.

## 1. Introduction

Multiscale techniques emerge from a class of problems where macroscopic continuum field variables fluctuate around a mean value at a small (Depending if small refers to a different order of magnitude or not, a different multiscale strategy is followed) window of length and/or time. This window is referred here as scale. The physics, chemists or mechanics which takes place at the microscale dictates the overall material behavior at the macroscale. Conversely, macroscopic effects on the specimen activate a cascade of events at the microscale which in turn evolves accordingly. Engineering design at the microscale is a powerful tool to get materials with improved mechanical, thermal, or electrical properties. Such a design is frequently inspired on nature, i.e., biomimetics. The different scales which may be identified in multiscale continuum problems, with to the problems of interest they are associated with, are introduced in the next sections.

Multiscale solvers have become the cornerstone in the so-called Integrated Computational Materials Engineering (ICME) philosophy, which is being rapidly adopted by the industry [1]. This new paradigm for the design of products comprises the analyses of several aspects of the materials used for a specific engineering application. Namely, their processing, the arrangement of their internal structure, their properties, and, finally, their (long-term) performance [2]. Obviously, such an approach requires the combination of several material models (i.e., physical phenomena) and multiple length (and time) scales in a seamless fashion. Therefore, multiscale solvers play a fundamental role in the virtual design and testing of materials [2,3].

### 1.1. Temporal Scales

Field variable fluctuations in the temporal scale are typically found in multiphysics problems, especially, in electromechanical ones. In this kind of problems, both electrical and mechanical waves are present, each of them with a different propagation velocity. Roughly (It depends on the physical and mechanical properties of the transmission material), electrical waves propagate at the speed of light, whereas mechanical wave velocity is of the order of the speed of sound. It is then clear the source of each temporal scale for these problems.

**Figure 1:**
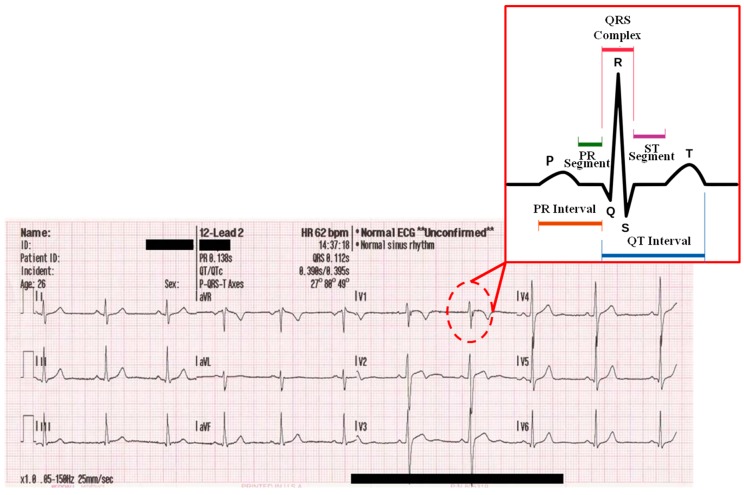
Electrocardiogram of a 26-year-old male (scale of the order of seconds). The electrical signal varies abruptly in a short period of time as detailed in the box (of the order of ms). A multiscale scheme considering both the fine temporal scale (ms) and the coarse one (s) is necessary to analyze the evolution of a cardiac disease, as an example, for long periods of time. Pictures taken from www.wikipedia.org.

A typical multiphysics example showing different time scales is heart electrophysiology (This problem also exhibits different hierarchical spatial scales. Here we only refer to the time scales). The electrical macroscopic activity of the heart, given in terms of an action potential electrical signal, is the phenomenological representation of the dynamic activity of ion channels that take place along the cardiomyocyte membrane [4]. Then, the network of cardiomyocytes present in the cardiac tissue is activated in a synchronized way by this electrical signal, giving place to a contraction of the tissue and finally releasing the blood ejection along the cardiovascular tree. Figure 1 shows a typical electrocardiogram highlighting the differences of time scales of the heart electrical activity. Multiscale modeling of heart electrophysiology is an extremely complex problem since it involves mechanical and electrical equations, as well as different temporal and hierarchical spatial scales. Several attempts have been made in this context to model electrophysiology in a multiscale fashion [5,6], and mechanoelectrical multiscale approaches [7], some of them integrated within the so-called ‘Virtual Physiology Human’ project [8].

### 1.2. Spatial Scales

**Figure 2:**
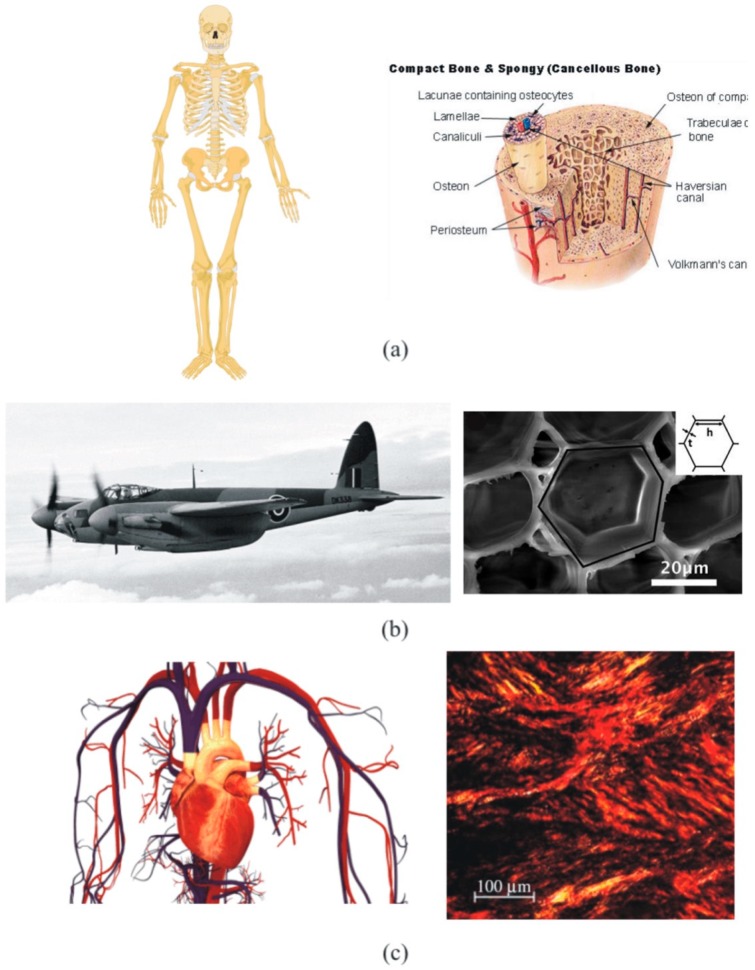
Examples taken from nature where different spatial scales can be distinguished. Left pictures are referred to the human (~m) scale (usually referred to as the ‘macroscopic’ or ‘coarse’ scale) while right ones correspond to a higher observation (~μm) scale (usually referred as the microscopic or finer scale): (**a**) human skeleton (left) and the typical microstructure of a flat bone (right). In this microstructure, one can visualize the engineering concept of a lightweight sandwich structure. The panels here refer to the cortical (low porosity) bone whereas the central zone is filled with cancellous (high porosity) bone; (**b**) World War II combat aircraft de Havilland Mosquito (left) and the microstructure (right) of its constituent materials (wood); (**c**) human vascular system (left) and microstructure of these soft fibered tissues showing the orientation of collagen fibers (right). All pictures taken from www.wikipedia.org except (**b**) right, taken with permission from [14].

Heterogeneous materials show a microstructure at a lower (or finer) observation spatial scale. This class of materials induces a fluctuation of the field variables in the finer scale of the order of the length of the heterogeneity. Examples of these materials include both natural and synthetic. Some examples of natural materials are enumerated as follows (see additionally Figure 2):

- Bone: Bone microstructure exhibits a certain degree of porosity ranging from 5% in cortical bone to 90% in cancellous or trabecular bone (see Figure 2a). Bones are part of the structural support of animals, i.e., the skeleton, so the criterion followed by evolution and natural selection in the design of such a microstructure is to economize the resistance/weight ratio. Man-made structures inspired by this criterion include the sandwich and foam structures. The associated multiscale problems in bone tissue are both mechanical and fluidics, since it is important to know the stress distribution and velocity of the fluid within the microstructure as well as the skeleton response to loads [9,10,11,12,13].
- Wood: This class of natural lightweight structures found in trees [14], see Figure 2b, is associated with structural problems with application in primitive handmade structures as well as light but stiff constructions, such as the World War II combat aircraft de Havilland Mosquito [15]. The microarchitectural arrangement of wood panels make them a lightweight stiff structure.
- Fibered tissues: Soft tissues are usually “reinforced” by collagen fibers (see Figure 2c). Examples of these tissues are blood vessels and arteries and the cardiac tissue, which is also embedded with cardiomyocytes. Collagen remodels in the microscopic scale during life tuning the mechanical properties of the macroscopic tissue, which influences the development of certain vascular disorders such as hypertension [16]. Even though the associated mechanical problem has been established macroscopically with success in the case of blood vessels [17,18,19,20,21], it is necessary to account for the fiber scale to include the mechanoelectrical activity of cardiomyocytes, as pointed out above, for the case of the cardiac tissue [22].

**Figure 3:**
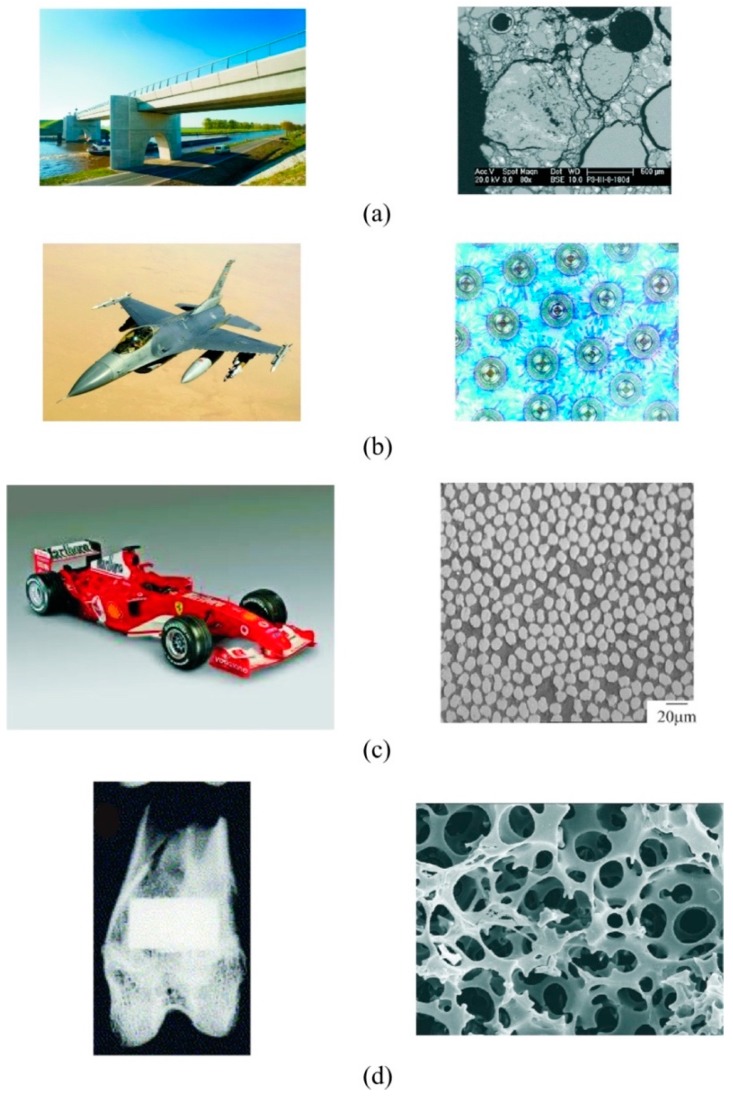
Artificial materials with a microstructure. Left pictures are referred to the human (~m) scale (usually referred as the macroscopic or coarse scale) while right ones correspond to a higher observation (~μm) scale (usually referred as the microscopic or finer scale). (**a**) (left) Concrete bridge (Picture taken from www.wikipedia.org) and (right) typical microstructure of a concrete building material (Picture taken with permission from [28]). (**b**) (left) F-16 Fighting Falcon aircraft. It uses monofilament silicon carbide fibers in a titanium matrix for a structural component of the jet’s landing gear (microstructure shown in right) (Picture taken from www.wikipedia.org). (**c**) (left) Ferrari F-1 prototype. Many parts of the structure are made of fiber carbon composite (Picture taken from www.wikipedia.org); right Microstructure of a carbon finer reinforced composite (Picture taken with permission from [34]). (**d**) (left) Bioceramic implant to promote new bone tissue regeneration in Tissue Engineering process. The implant is microstructurally featured in right. It is a porous scaffold where cells attach, segregate new matrix and finally new bone tissue regeneration. (Picture (**d**) (left) taken with permission from [35] and (right) taken with permission from [36]).

On the other hand, synthetic materials are provided with an artificial microstructure, composed of different phases, in order to get improved mechanical, thermal, or electrical properties that each raw material itself cannot reach alone. Many of the artificially designed microstructural materials are bio-inspired to mimic some of the extraordinary properties of natural materials highlighted above. Examples of these materials are composites, alloys, or biomaterials among many others. Some of them are listed below (see additionally Figure 3):

- Concrete: It has been widely used as a building material since the mid-18th century. It is microstructurally made through a mixture of water, cement, aggregates, and reinforcement. The result is a cheap, easy, and resistant macroscopic material (see Figure 3a). The overall mechanical and thermal behavior can be obtained by an analysis of its microstructure. Regarding its mechanical behavior, it is well known that, microscopically, the progression of cracks in the cement is stopped by the aggregates, whereas the reinforcement provides tension stiffness. Recently, engineered cementitious composites (ECC) have emerged as a class of ultra-ductile fiber-reinforced cementitious composites, whose better mechanical properties were the result of years of study to develop a material microstructurally designed using micromechanics concepts [23].
- Metal matrix composites (MMC): They belong to the class of composite materials with different phases being one of them at least a metal. A two-phase MMC contains a matrix and a reinforcement. The idea is to obtain a hybrid material with excellent properties such as wear resistance, friction coefficient, mechanical resistance, or thermal conductivity (see Figure 3b). All these properties can be derived from an analysis of its associated structure at the microstructural level.
- Composite materials: These are fibered materials usually composed of a polymeric or resin matrix phase and a fiber reinforcement (see Figure 3c). They have excellent resistance/weight ratio as well as electrical, thermal and acoustic isolating properties. All these properties derive from the microscopic orientation and density of fibers within the matrix.
- Biomaterials: The current generation of biomaterials includes self-active materials which interact with the human body with improved regeneration and healing capabilities. An example is the scaffolds used in tissue engineering. Here scaffolds are used as a temporary porous structural support to attach cells and to segregate new matrix tissue. After the regeneration process is complete the structure naturally degrades (see Figure 3d). The associated analysis of this problem is both multiscale and multiphysical in nature. A summary of it may be found in [24].

### 1.3. Scope and Outline

This paper reviews the state-of-the-art of multiscale solvers in continuum problems. The authors are aware there exist a number of review papers on these topic (see for example [25,26,27] among many others). However, these papers are generally focused on multiscale mechanics (natural extensions to geometric and material nonlinear mechanics are of course included within these papers) with a lack of motivation of problems with a technological interest solved via a multiscale technique. These technological problems are not necessarily posed in a mechanical context but thermal, diffusive, fluidic, or a multiphysical combination of them. Moreover, some of those papers are bounded to computational homogenization techniques. In this paper, we review the proper mathematical definition associated with a number of continuum problems and their multiscale strategy of solution. Indeed, several multiscale schemes, not only those based on computational homogenization, are reviewed. However, we do not go into detail in the review of the rigorous mathematics—i.e., proofs or theorems—behind each multiscale technique. The current literature on multiscale problems is vast, so we restrict ourselves in this paper to continuum problems both at the macro and microstructural levels. It is then out of the scope of this paper the review of multiscale techniques based on atomistic modeling and coarse-graining strategies. The reader is referred to [28,29,30,31,32,33] which address and review these topics. The paper is organized as follows. First, we review a number of multiscale continuum problems based on the homogenization technique. The technological application and interest of each problem is emphasized. Second, we review some other multiscale problems which do not follow the homogenization scheme and are then classified as non-homogenization methods. Next, we review new trends in computational multiscale schemes, such as those based on proper generalized decomposition (PGD). Finally, a summary of existing software for the implementation of the aforementioned numerical schemes is presented. 

## 2. Homogenization-Based Multiscale Approaches

In multiscale problems one faces the macroscale where the macroscopic domain is defined, and the microscale where the material heterogeneities and microstructural details can be visualized. If $\ell_{m}$ is the typical size of the heterogeneity and $\ell_{M}$ the typical size of the macroscale, then we assume there exists separation of scales if $\ell_{M}\gg \ell_{m}$. Under this principle, homogenization-based multiscale (HM) approaches are feasible.

**Figure 4:**
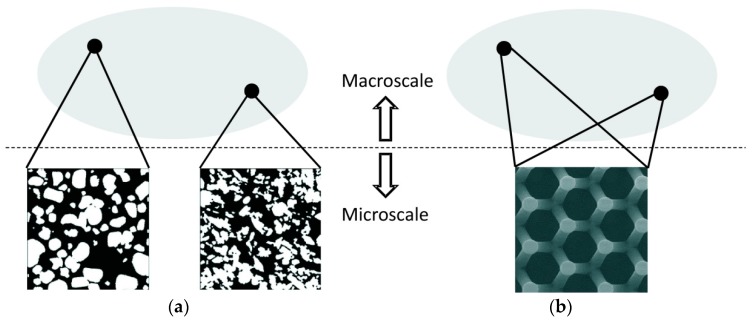
Concept of local (**a**) and global (**b**) periodicity. (Adapted from figures taken with permission from [38,39]).

The first step in HM is the definition of the domain of the microstructure. It is defined in terms of a so-called representative volume element (RVE) which represents the underlying microstructure in the neighborhood of a certain macro point [37]. An RVE must contain enough statistical information about the inhomogeneous medium to be representative of the material itself. This RVE may represent the whole macro-domain (global periodicity) or not (local periodicity), see Figure 4. A critical issue in HM is the determination of the RVE size ($\ell_{RVE}$). A priori, $\ell_{RVE}\gg \ell_{m}$, although it depends on the degree of nonlinearity of the equations to be solved in the microscale, or differences on the properties of the micro-constituents [38,39]. In the limit case, i.e., $\ell_{RVE}\sim \ell_{M}$, there is not an actual RVE. In fact, the RVE existence is not always assured (see an analysis on the RVE existence of quasi-brittle heterogeneous materials in [40]).

**Figure 5:**
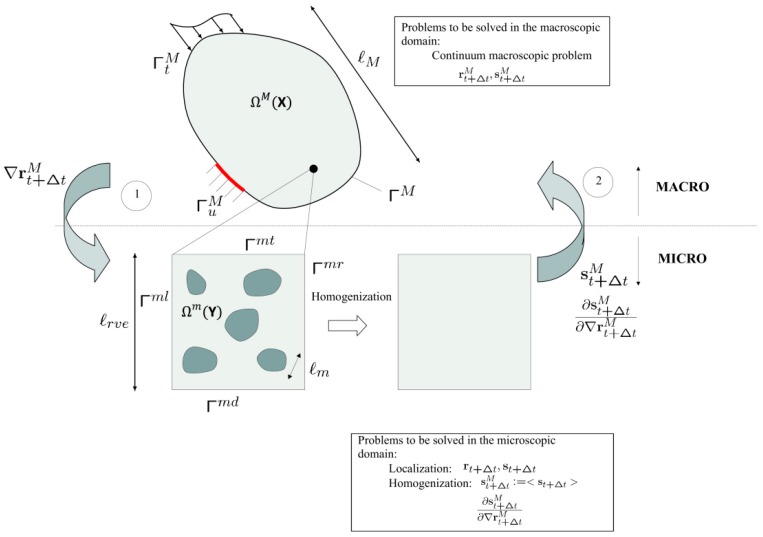
General multiscale procedure based on first order homogenization. Field and state variables computed at the macroscale for a given time increment are denoted as $r_{t+\Delta t}^{M}$ and $s_{t+\Delta t}^{M}$, respectively. The first gradient of the field variable $\nabla r_{t+\Delta t}^{M}$ is provided to the microscopic scale. Then, the localization problem is solved and the microscopic quantities $r_{t+\Delta t}$ and $s_{t+\Delta t}$ are obtained. Finally, the homogenized variable $s_{t+\Delta t}^{M}$ and the linearized material tangent operator $\partial s_{t+\Delta t}^{M}/\partial \nabla r_{t+\Delta t}^{M}$ are passed to the macroscopic scale. This process is repeated iteratively.

The general procedure followed in HM is shown in Figure 5. Briefly, and for a generic problem, for a certain time step t+Δt, the first gradient $\nabla r_{t+\Delta t}^{M}$ of the field (scalar or vectorial) macroscopic variable $r_{t+\Delta t}^{M}$ is given in the microscopic domain. There, the associated localization problem is solved, and the averaged (vectorial or tensorial) state variables $s_{t+\Delta t}^{M}\;:\langle s_{t+\Delta t}\rangle$ are homogenized. The state variables are obtained through the constitutive relationships of the microconstituents of the microscopic domain, such that, $s_{t+\Delta t}=f\left(\nabla r_{t+\Delta t},\nabla {\dot{r}}_{t+\Delta t},\ldots \right)$. Hereafter, M and m denote the macroscopic and microscopic scales, respectively, whereas the averaging symbol is defined as

(1) $$\langle \cdot \rangle =\frac{1}{\left|\Omega^{m}\right|}\int_{\Omega^{m}}\cdot dY$$

with $\Omega^{m}$ the volume of the microscopic domain. We use X and Y to define a material point at the macroscopic and microscopic scale, respectively. There are many variations of this general procedure depending on the particularities of the problem at hand. The differences from this general method are illustrated for each specific problem below.

Since in HM methods the micro domain (RVE) generally does not physically represent the exact underlying geometry of its macroscopic location, boundary conditions at the microscale are not trivially defined. In classical approaches, Neumann, Dirichlet, or periodic boundary conditions are usually imposed. They are summarized below:

- Neumann: ${\bar{t}}_{t+\Delta t}=s_{t+\Delta t}^{M}\left(x\right)\cdot n^{m}\left(Y\right),Y\in \Gamma^{m}$
- Dirichlet: ${\bar{r}}_{t+\Delta t}=\nabla r_{t+\Delta t}^{M}\left(X\right)\cdot Y,Y\in \Gamma^{m}$
- Periodic: ${\bar{r}}_{t+\Delta t}^{\Gamma^{ml}}={\bar{r}}_{t+\Delta t}^{\Gamma^{mr}}={\bar{r}}_{t+\Delta t}^{\Gamma^{mt}}={\bar{r}}_{t+\Delta t}^{\Gamma^{md}},\Gamma^{ml},\Gamma^{mr},\Gamma^{mt},\Gamma^{md}\in \Gamma^{m}$

being $n^{m}$ the outward normal of the microscopic RVE, $\Gamma^{m}$ the microscopic boundary; $\Gamma^{ml},\Gamma^{mr},\Gamma^{mt},\Gamma^{md}$ the left, right, top, and down parts of the boundary $\Gamma^{m}$ (see Figure 5). Even though if the microstructural domain is non-periodic, periodic boundary conditions generally provide the most accurate approach in HM computations [41].

The process shown in Figure 5 is repeated iteratively. The downscaling problem is called the localization problem, whereas upscaling is known as homogenization. These issues are addressed in the following sections for different problems of industrial interest.

### 2.1. Linear Elasticity

Linear elastic problems with a microstructure cover a wide range of technological problems such as those as heterogeneous materials, e.g., fibered composites, porous materials, concrete, metal matrix composites, or reinforced materials with different microstructural phases. The general setup of HM presented above is not followed in linear elastic problems, but they are focused on the computation of the averaged macroscopic or apparent properties (directly computed from the microstructure) and obtaining the canonical functions available to solve the localization problem at the microstructure. This methodology is reviewed below.

Classical literature on micromechanics has analyzed this problem since the seminal work of Eshelby [42]. Several analytical theories have emerged to compute the apparent or effective mechanical properties tensor ${\mathbb{C}}^{M}$, such as the asymptotic homogenization theory, and the effective properties of materials with a simple regular microstructure have been analytically estimated [41,43,44,45,46]. Next, the localization and homogenization problems are introduced in the framework of linear elastic problems.

#### 2.1.1. Localization

Based on the asymptotic splitting of the displacement field $u\left(X,Y\right)=u^{M}\left(X\right)+u^{m}\left(Y\right)$, the linear, elastic, microscopic problem in the absence of body loads reads as,

(2) $$\begin{array}{c} \nabla \cdot \sigma =0\\ \varepsilon =\frac{1}{2}\left(\nabla u+\nabla u^{T}\right)\\ \sigma ={\mathbb{C}}^{M}:\varepsilon \\ \langle \varepsilon \rangle =\varepsilon^{M} \end{array}$$

ε and σ being the (linearized) strain and stresses state variables in the microstructure, and ${\mathbb{C}}^{M}$ the linear elastic fourth order tensor containing the mechanical properties of the micro-constituents of the microstructure.

The absence of boundary conditions is noticeable on Equation (2). These boundary conditions must reproduce, as closely as possible, the in-situ state of the RVE inside the material. Therefore, they strongly depend on the choice of the RVE itself, and specially on its size. Regardless whether periodic media are under consideration or not, the periodicity conditions are assumed, as discussed above, which imply the following statements:

1. The stress vectors $\sigma \cdot n^{m}$ are opposite on opposite sides of the boundary $\Gamma^{m}$.
2. The local strain ε(u) is split into its average and fluctuating terms such that,
  (3) $$\begin{array}{c} \varepsilon \left(u\right)=\varepsilon^{M}+\varepsilon^{m}\left(u^{m}\right)\\ \langle \varepsilon^{m}\left(u^{m}\right)\rangle =0 \end{array}$$
  thus, the periodicity boundary conditions yield
  (4) $$\begin{array}{c} \sigma^{m}\cdot n^{m}\;\mathrm{anti}-\mathrm{periodic}\;\mathrm{on}\;\Gamma^{m}\\ u=\varepsilon^{M}\cdot Y+u^{m}\;\mathrm{on}\;\Gamma^{m}\\ u^{m}\;\mathrm{periodic}\;\mathrm{on}\;\Gamma^{m} \end{array}$$

By virtue of the linearity of the problem, the solution of $\varepsilon^{m}\left(u^{m}\right)$ in Equation (2) for a general macro strain $\varepsilon^{M}$ may be expressed as the superposition of elementary unit strain solutions $\varepsilon^{m}\left(\chi_{kh}\right)$ [41], such as,

(5) $$\varepsilon^{m}\left(u^{m}\right)=\varepsilon_{kh}^{M}\varepsilon^{m}\left(\chi_{kh}\right)$$

where $\chi_{kh}$ are the displacements associated with those elementary strain states (canonical functions) denoted by indices kh resulting from the solution of Equation (2).

By substitution of Equation (5) into Equation (3), the micro-strains are expressed as

(6) $$\varepsilon^{m}\left(u^{m}\right)=\varepsilon_{kh}^{M}\left(I_{kh}+\varepsilon^{m}\left(\chi_{kh}\right)\right)$$

where $I_{kh}$ is the identity fourth-order tensor with components $I=I_{kh}={\left(I_{ij}\right)}_{kh}=\frac{1}{2}\left(\delta_{ij}\delta_{jh}+\delta_{ih}\delta_{jk}\right)$.

Note that the above analysis has been accomplished taking into account that $\varepsilon^{M}$ is given by the macroscopic scale. A similar approach may be established when $\sigma^{M}$ is given (see [41] for details). Once computed the canonical functions $\chi_{kh}$ (which are only a function of the microstructural geometry), the strain and stress state at the microstructural level can be directly computed for any given macroscopic strain state $\varepsilon^{M}$.

#### 2.1.2. Homogenization

Once the elementary solutions $\varepsilon^{m}\left(\chi_{kh}\right)$ are obtained, the macroscopic stress-strain relationship is obtained straightforwardly,

(7) $$\sigma^{M}=\langle \sigma \left(u\right)\rangle =\langle {\mathbb{C}}^{m}:\varepsilon \left(u\right)\rangle =\langle {\mathbb{C}}^{m}\left(I_{kh}+\varepsilon^{m}\left(\chi_{kh}\right)\right)\rangle :\varepsilon^{M}$$

Consequently, at the macroscopic scale, the elasticity tensor is identified in Equation (7) as,

(8) $${\mathbb{C}}^{M}=\langle {\mathbb{C}}^{m}\left(I_{kh}+\varepsilon^{m}\left(\chi_{kh}\right)\right)\rangle$$

#### 2.1.3. Variational Formulation

To get a finite element implementation of the problem above, Equations (2) and (3) are used in order to write the variational form, namely,

(9) $$\int_{\Omega^{m}}\varepsilon^{m}\left(w\right):\left({\mathbb{C}}^{m}:\varepsilon^{m}\left(u^{m}\right)\right)dY=-\int_{\Omega^{m}}\varepsilon^{m}\left(w\right):\left({\mathbb{C}}^{m}:\varepsilon^{M}\right)dY\;\forall w\left(Y\right)\in V_{Y}$$

where the space $V_{Y}$ is defined as,

(10) $$V_{Y}=\left\{w\;|w\in H^{1}\left(\Omega^{m}\right)\right\}$$

with $H^{1}\left(\Omega^{m}\right)$ the first-order Sobolev space. Using Equations (5) and (9) can be further developed, namely,

(11) $$\int_{\Omega^{m}}C_{ijpq}^{m}\frac{\partial \chi_{p}^{kl}}{\partial y_{q}}\frac{\partial \omega_{i}}{\partial y_{j}}dY=\int_{\Omega^{m}}C_{ijkl}^{m}\frac{\partial \omega_{i}}{\partial y_{j}}dY$$

where $\chi_{p}^{kl}$ represents the characteristic microstructure displacement at p directions due to an applied kl unit strain, being k=l normal unit strain states and k≠l shear unit strain states. $\omega_{i}$ represents a virtual displacement. The total strain states are six (three normal and three shear) corresponding to the six linear equations above (Equation (11)). Once these functions are obtained, the macroscopic stiffness tensor can be computed through Equation (8).

#### 2.1.4. Illustrative Example

**Figure 6:**
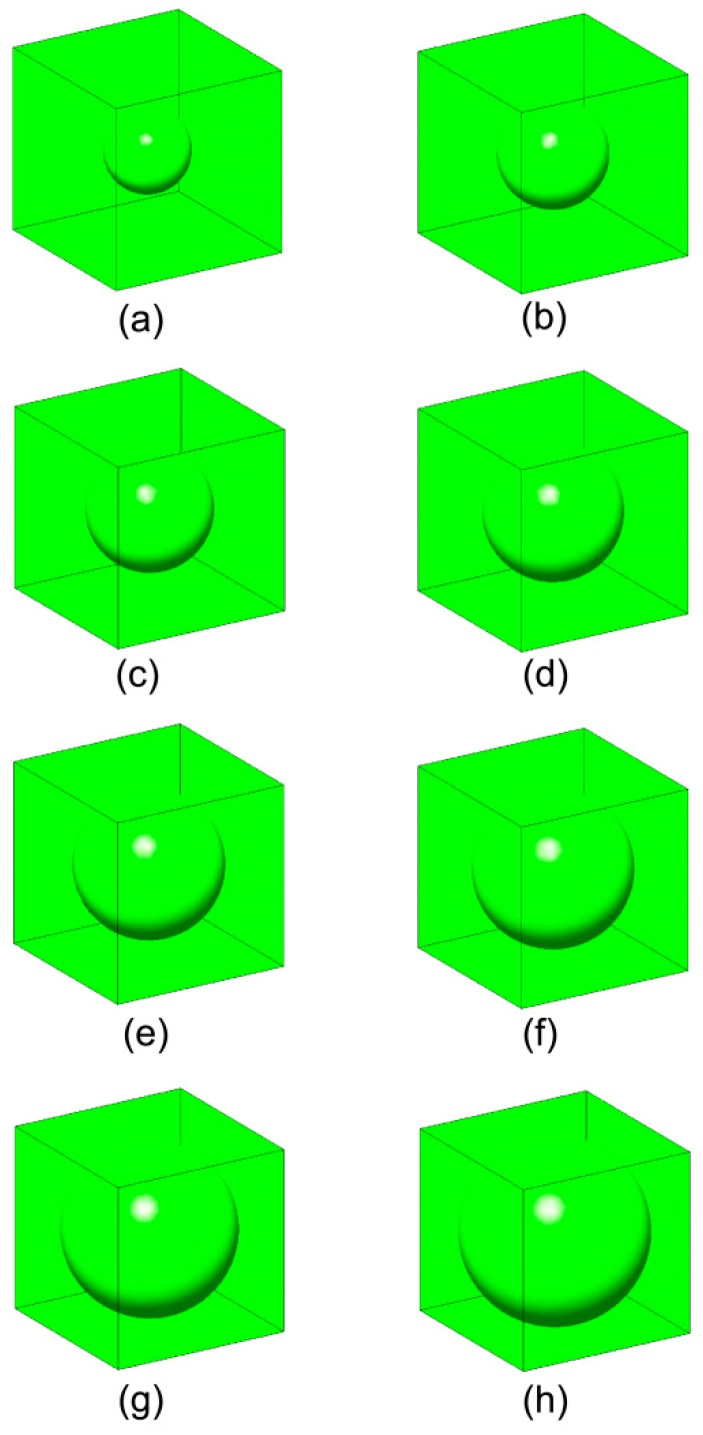
Solid cube with spherical void inclusion for mechanical properties homogenization. Void fractions (**a**) 0.05; (**b**) 0.1; (**c**) 0.15; (**d**) 0.2; (**e**) 0.25; (**f**) 0.3; (**g**) 0.4; (**h**) 0.5.

Results of the implementation of the computational homogenization theory presented above are shown over a periodic medium containing spherical voids. The overall effective moduli of this microstructure are expressed as a function of the void fraction. The theoretical estimates of the homogenized mechanical properties were presented in [45]. Thus, a unit cell containing different fractions of voids was modeled (see Figure 6).

**Figure 7:**
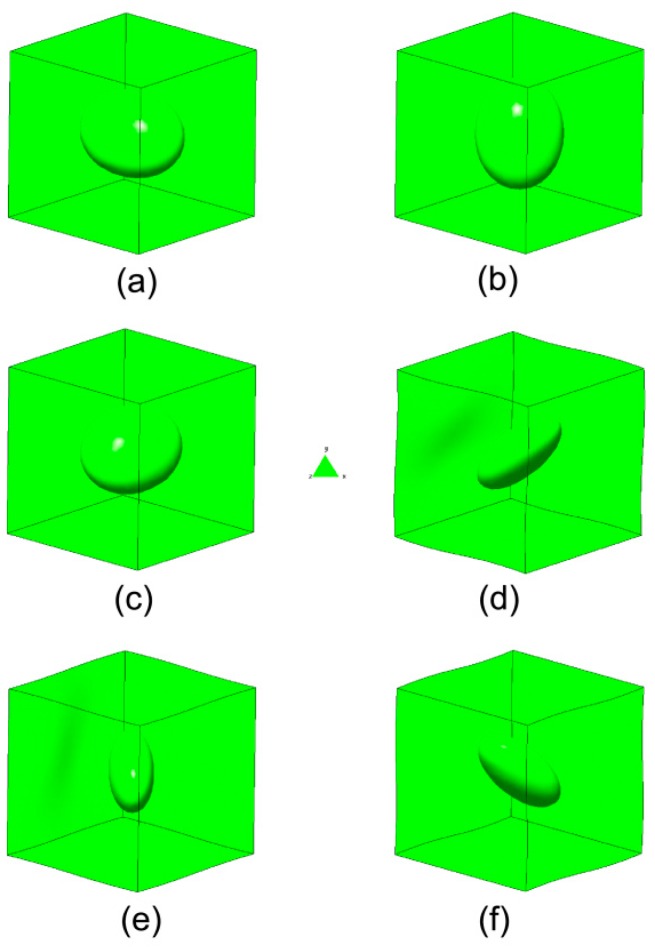
Characteristic deformations of solid cube with spherical void inclusion (0.05). (**a**) $\chi^{11}$; (**b**) $\chi^{22}$; (**c**) $\chi^{33}$; (**d**) $\chi^{12}$; (**e**) $\chi^{13}$; (**f**) $\chi^{23}$.

**Figure 8:**
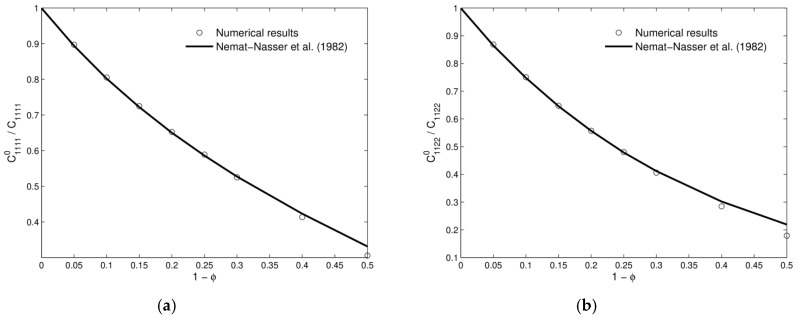
Theoretical estimates [47] versus numerical values of effective elastic moduli of a body with spherical voids (void fraction ϕ). (**a**) $C_{1111}^{M}/C_{1111}^{m}$; (**b**) $C_{1122}^{M}/C_{1122}^{m}$.

The RVE was subjected to unit strain states and the characteristic deformations are plotted in Figure 7. A comparison of the obtained results versus the theoretical estimates presented in [45] is shown in Figure 8 and Table 1. A good agreement was found.

The presented results show the ability of this technique to derive effective properties directly from materials microstructure. It is also useful for the study of the influence of the microstructure on the degree of anisotropy of a certain micro-architectural design or the derivation of macroscopic properties of concrete [47,48,49], ceramics [50], and composites [51,52,53,54,55,56].

### 2.2. Nonlinear Mechanics

Nonlinear mechanics multiscale formulation has to be established for every time step and macropoint, since the overall macroscopic behavior is dictated by the microstructure evolution in terms of internal variables evolution, which in turn, depend on the macroscopic history load. Therefore, the multiscale strategy of solution yields the so-called FE^2^ scheme [57]. The process is roughly illustrated in Figure 2. We distinguish in this section between material and geometrical nonlinearities.

A generic material nonlinear problem at small strains, and considering the asymptotic splitting of the displacement field $u\left(X,Y\right)=u^{M}\left(X\right)+u^{m}\left(Y\right)$, is modeled as follows,

(12) $$\begin{array}{c} \nabla \cdot \sigma =0\\ \varepsilon =\varepsilon^{M}\left(u^{M}\right)+\varepsilon^{m}\left(u^{m}\right)\\ \sigma =f\left(\varepsilon ,\overset{˙}{\varepsilon },\Theta_{i},\ldots \right)\\ \langle \varepsilon \rangle =\varepsilon^{M}\\ \langle \sigma \rangle =\sigma^{M}\\ +\;\mathrm{boundary}\;\mathrm{conditions} \end{array}$$

Note that the stress tensor is evaluated in Equation (12) once defined its nonlinear constitutive relationship (plasticity, damage, etc.) which may eventually be a function of the strain and strain rate, and of internal variables denoted as $\Theta_{i}$. The strain operator is defined as,

(13) $$\varepsilon \left(\cdot \right)=\frac{1}{2}\left(\nabla \cdot +\nabla \cdot^{T}\right)$$

Macroscopically, the variational formulation of the problem yields,

(14) $$\int_{\Omega^{M}}\sigma^{M}:\varepsilon^{M⋆}dX=\int_{\Gamma_{t}^{M}}{\bar{t}}^{M}\cdot u^{M⋆}dX$$

where $u^{M⋆}$ represents a compatible virtual displacement (trial) function defined at the macroscale, and according to (13), $\varepsilon^{M⋆}=\varepsilon^{M⋆}\left(u^{M⋆}\right)$. Using (12) in (14) yields,

(15) $$\int_{\Omega^{M}}\left[\frac{1}{\left|\Omega^{m}\right|}\int_{\Omega^{m}}f\left(\varepsilon^{M}+\varepsilon^{m},{\overset{˙}{\varepsilon }}^{M}+{\overset{˙}{\varepsilon }}^{m},\Theta_{i},\ldots \right)dY\right]\;:\varepsilon^{M⋆}dX=\int_{\Gamma_{t}^{M}}{\bar{t}}^{M}\cdot u^{M⋆}dX$$

Microscopically, the variational formulation of the problem, considering the periodicity conditions, yields,

(16) $$\int_{\Omega^{M}}\sigma :\varepsilon^{m⋆}dY=0$$

Again, substitution of (12) in (16) yields,

(17) $$\int_{\Omega^{m}}f\left(\varepsilon^{M}+\varepsilon^{m},{\overset{˙}{\varepsilon }}^{M}+{\overset{˙}{\varepsilon }}^{m},\Theta_{i},\ldots \right)\;:\varepsilon^{M⋆}dY=0$$

Equations (15) and (17) represent a set of general integral differential equations, which couples the macro and the micro scales. The reader is referred to [25] for the specific derivation of such equations for a variety of material nonlinear problems.

The finite element implementation of the multiscale nonlinear problem requires linearization of Equations (15) and (17) of the specific problem at hand. Linearization induces an FE^2^ solution strategy. References [25,57,58] include a number of FE^2^ multiscale nonlinear material problems and their numerical implementation.

**Figure 9:**
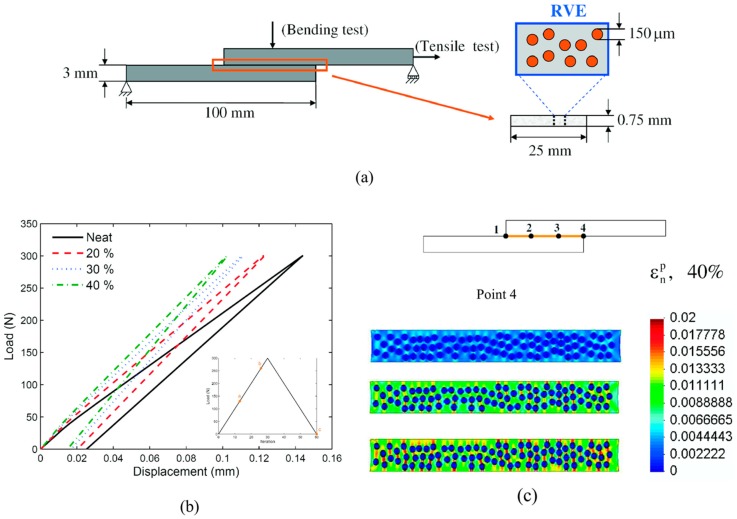
Multiscale analysis of heterogeneous adhesives. (**a**) Bending test macroscopic setup of a microstructurally reinforced adhesive. (**b**) Macroscopic traction-separation law in the normal direction, based on a multiscale analysis, for different concentrations of the inclusion and the bending loading included in the inset. (**c**) Microstructural distribution of the normal plastic microstrains for a 40% concentration of inclusions at the right corner side of the adhesive (point 4), at the three first stage levels of the macroscopic loading (inset in (**b**)). (Figures taken with permission from [61]).

Interestingly, the multiscale formulation above has been particularized for heterogeneous adhesives in [59,60,61]. This problem has many applications since many aerospace, aircraft or automotive mechanical components are joined together by using a structural reinforced (heterogeneous) adhesive. At the macroscale, the adhesive behavior is featured by a cohesive zone modeling approach, whereas microstructurally the localization and homogenization problems are solved, which dictates the overall traction-separation macroscopic law. In [59] the matrix of the microstructure is modeled through a classical damage approach, and in [61] the matrix of the adhesive is considered to obey an elastic-plastic behavior. Figure 9a shows the bending test of a particle-reinforced heterogeneous adhesive. It can be seen in Figure 9b the macroscopic (normal) traction-separation law for different concentration of inclusions, obtained using the multiscale approach. Figure 9c represents the distribution of the plastic normal micro strain throughout the microstructure of the macro point 4. The modeling here presented shows the ability of multiscale techniques to analyze complex adhesive microstructure including the behavior of both matrix and inclusion.

On the other hand, microscopic (static) equations of geometrically nonlinear materials, in the absence of body forces, are written as (material version):

(18) $$\begin{array}{c} \nabla_{X,Y}\cdot P=0\\ P=f\left(F,\dot{F},\Theta_{i},\ldots \right)\\ \langle F\rangle =F^{M}\\ \langle P\rangle =P^{M}\\ +\;\mathrm{boundary}\;\mathrm{conditions} \end{array}$$

being P and $P^{M}$ the microscopic and macroscopic first Piola–Kirchhoff stress tensor, respectively. This stress tensor is evaluated in Equation (18) once defined its (hyperelastic) constitutive relationship of the microstructure (finite strain plasticity, damage, etc.) which may eventually be a function of the deformation gradient F and its rate $\dot{F}$, and of internal variables denoted as $\Theta_{i}$. The deformation gradient is defined as,

(19) $$F=\nabla_{X,Y}x$$

Moreover, the Hill–Mandel condition or macro-homogeneity condition [36,40] read as,

(20) $$\frac{1}{\left|\Omega^{m}\right|}\int_{\Omega^{m}}P:\delta FdY=P^{M}:\delta F^{M}$$

Now, all the ingredients have been introduced in order to develop the multiscale weak form of the geometrically nonlinear problem above. The macroscopic first variation of the weak form is written as follows (material version),

(21) $$\int_{\Omega^{M}}P^{M}:\delta F^{M⋆}dX=\int_{\Gamma_{t}^{M}}{\bar{T}}^{M}\cdot \delta u^{M⋆}dX$$

where $\delta u^{M⋆}$ represents a compatible virtual displacement (trial) functions defined at the macroscale, and its associated deformation gradient $\delta F^{M⋆}$. Using (18) in (21), we get

(22) $$\int_{\Omega^{M}}\left[\frac{1}{\left|\Omega^{m}\right|}\int_{\Omega^{m}}f\left(F,\dot{F},\Theta_{i},\ldots \right)dY\right]:\delta F^{M⋆}dX=\int_{\Gamma_{t}^{M}}{\bar{T}}^{M}\cdot \delta u^{M⋆}dX$$

Microscopically, the variational formulation of the problem, considering the periodicity boundary conditions, yields,

(23) $$\int_{\Omega^{M}}P^{M}:\delta F^{M⋆}dY=0$$

Again, using (18) in (23), we can write

(24) $$\int_{\Omega^{m}}f\left(F,\dot{F},\Theta_{i},\ldots \right):\delta F^{m⋆}dY=0$$

Equations (22) and (24) represent a set of general integral differential equations, which couples the macro and the micro scales in geometrically nonlinear systems. As in material nonlinearities, this procedure takes advantage of FE^2^ solutions.

**Figure 10:**
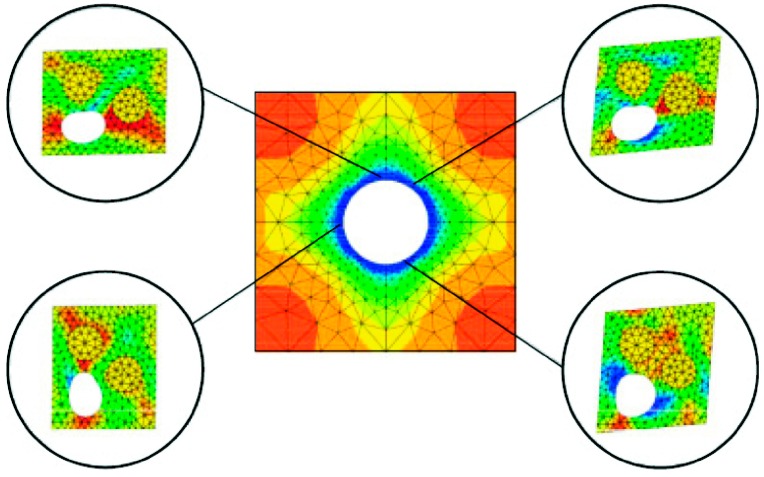
Multiscale analysis of finite strain plasticity: Equivalent plastic strains (figure taken with permission from [26]).

The reader is referred to [26], and references therein, for a review on the formulation and finite element implementation of geometrically nonlinear multiscale problems. Figure 10 represents both macro and microscopic equivalent plastic strains of a multiscale formulation of a finite strain plasticity problem.

The geometrically nonlinear multiscale procedure outlined above is based on a first order homogenization scheme. Nonetheless, it is based on the sketch presented in Figure 2 being in this figure the macroscopic variable given to the microscopic scale $F_{t+\Delta t}^{M}$, and the homogenized variable available at the macroscale $P_{t+\Delta t}^{M}$ This methodology lacks of accuracy (both quantitative and qualitative) in problems undergoing large gradients of the deformation field, i.e., localization problems or another class of problems where size effects in the microstructure are relevant [62,63]. These kinds of problems arise in materials which undergoes both softening and hardening behavior. In this context, a second order homogenization approach has been proposed in [64]. Briefly, in this work the authors propose to pass to the microstructure both the first and second gradients of the deformation field (see Figure 2). The inconvenience of this solution is writing a new equilibrium equation at the macroscale associated with the homogenized second gradient (see [64] for details).

Another problem encountered in nonlinear multiscale problems is the loss of convexity of the macroscopic strain energy density function which is computed and homogenized ‘on the fly’ during the multiscale analysis. This shortcoming appears in problems showing loss of stability (bifurcation) in the microstructure, or those including discontinuities, such as cracks, in the microstructure. The former class of problems was studied in [65] using Γ-convergence available for non-convex potentials. Moreover, in this work the representativeness of the RVE for this kind of problems was determined by means of bifurcation theory. On the other hand, microstructure with discontinuities and associated numerical problems were dealt in [66,67] using the multiscale aggregating method and related works [68,69].

### 2.3. Darcy and Fick Problems

Heterogeneous materials with microstructure are also interesting for fluidic and diffusive processes in applications such as filters, viscous dampers, catalyzers, or tissue engineering scaffolds. Here the (linear) problem to analyze is based on the Darcy’s law of liquid percolation, which is also analogous to the Fick’s law for diffusion. Both problems are then mathematically analogous, as well as the interpretation of the permeability and diffusion tensors for Darcy’s and Fick’s based phenomena.

In the following sections, the multiscale problem associated with the linear steady-state flow motion within a microstructure is presented.

#### 2.3.1. Localization

Like in the linear elastic case, the microstructural flow motion of a liquid at the microscale is given by solving the associated localization problem, which in turn can be computed once the canonical functions of the microstructural velocities are computed. Then, the macroscopic associated problem, i.e., Darcy’s law, is featured by the permeability tensor directly derived from the underlying porous microstructure.

Following the asymptotic homogenization theory, we expand the velocity and pressure fields, v and p, as,

(25) $$\begin{array}{c} v\left(X,Y\right)=v^{M}\left(x\right)+v^{m}\left(Y\right)\\ p\left(X,Y\right)=p^{M}\left(x\right)+p^{m}\left(Y\right) \end{array}$$

Then, the equilibrium and continuity microscopic equations for an incompressible viscous fluid within the steady state Stokes flow problem read as,

(26) $$\begin{array}{c} -\nabla p\cdot I+\mu \nabla \cdot \nabla v+\rho f=0\\ \nabla \cdot v=0\\ v=0\in \Gamma^{m}\;(\mathrm{no}-\mathrm{slip}\;\mathrm{condition})\\ +\mathrm{boundary}\;\mathrm{conditions} \end{array}$$

with ρ and μ being the fluid density and dynamic fluid viscosity, respectively. The body forces vector per unit volume is denoted by f. For brevity, we define Z=−∇p·I+ρf, with I the second-order identity tensor. Since we deal with periodic boundary conditions, we establish an analogous procedure to (4) for the fluid problem,

(27) $$\begin{array}{c} p^{m}n^{m}\;\mathrm{antiperiodic}\;\mathrm{on}\;\Gamma^{m}\\ v^{m}\;\mathrm{periodic}\;\mathrm{on}\;\Gamma^{m} \end{array}$$

As in the solid problem, the micro-velocities in (26) allow a solution of the form,

(28) $$v^{m}=-Z_{j}^{m}\kappa_{j}^{i}$$

with $\kappa_{j}^{i}$ the characteristic fluid velocities associated with unit pressure gradients [70]. Equation (28) allows to obtain the microvelocities along the microscopic domain subjected to a macroscopic pressure $Z^{m}$.

After some algebraic manipulations and using (25), (26), and (28), the functions $\kappa_{j}^{i}$ [70] must obey the following expression in the microscopic fluid domain of the RVE,

(29) $$\mu \nabla^{2}\kappa =-Z^{m}$$

in combination with the boundary conditions (27).

#### 2.3.2. Homogenization

To obtain the homogenized macroscopic Darcy’s law, the microvelocities in Equation (28) are averaged over the microstructural domain (RVE) as follows,

(30) $$K=\langle \kappa_{j}^{i}\rangle$$

with K being the macroscopic permeability matrix. To derive Equation (30), the null fluctuation of the velocity field $\langle v^{m}\rangle =0$ in $\Omega^{m}$ has been taken into consideration.

#### 2.3.3. Variational Formulation

The variational form of the fluid phase is developed similarly to the case of solids. Therefore, by using the periodicity assumptions in Equation (27) the variational form of Equation (26) yields to,

(31) $$\mu \int_{\Omega^{m}}\nabla \gamma \cdot \nabla v^{m}dY=-\int_{\Omega^{m}}Z^{M}\cdot \gamma dY\;\forall \gamma \left(Y\right)\in V_{Y}$$

being the space $V_{Y}$ defined as in the section above. Using the superposition of Equation (28) and the result Equation (29) we finally get the following integral expression,

(32) $$\mu \int_{\Omega_{F}^{m}}\frac{\partial \kappa_{i}^{j}}{\partial y_{p}}\frac{\partial \gamma_{i}^{j}}{\partial y_{p}}dY=\int_{\Omega^{m}}\gamma_{j}dY$$

where $\kappa_{i}^{j}$ represents the characteristic microstructure fluid velocity due to an applied generalized pressure gradient $Z_{i}^{M}$ in the direction i and $\gamma_{j}$ represents a virtual velocity. Once these functions are obtained, the macroscopic permeability $K_{ij}$ may be obtained from Equation (30).

#### 2.3.4. Illustrative Example

The developed homogenization modeling presented for fluids is applied to a periodic microstructure composed by a cylinder void immersed within a fluid medium.

This problem has many interests in composite manufacturing, since the cylinder void may reproduce the composite fibers to model intra-yarn flow in permeable yarns, being the permeability a critical parameter in this process. In fact, the mathematical determination and prediction of the permeability tensor for these applications have been an active field of research in the last decade [71,72,73,74,75,76,77,78,79,80,81].

**Figure 11:**
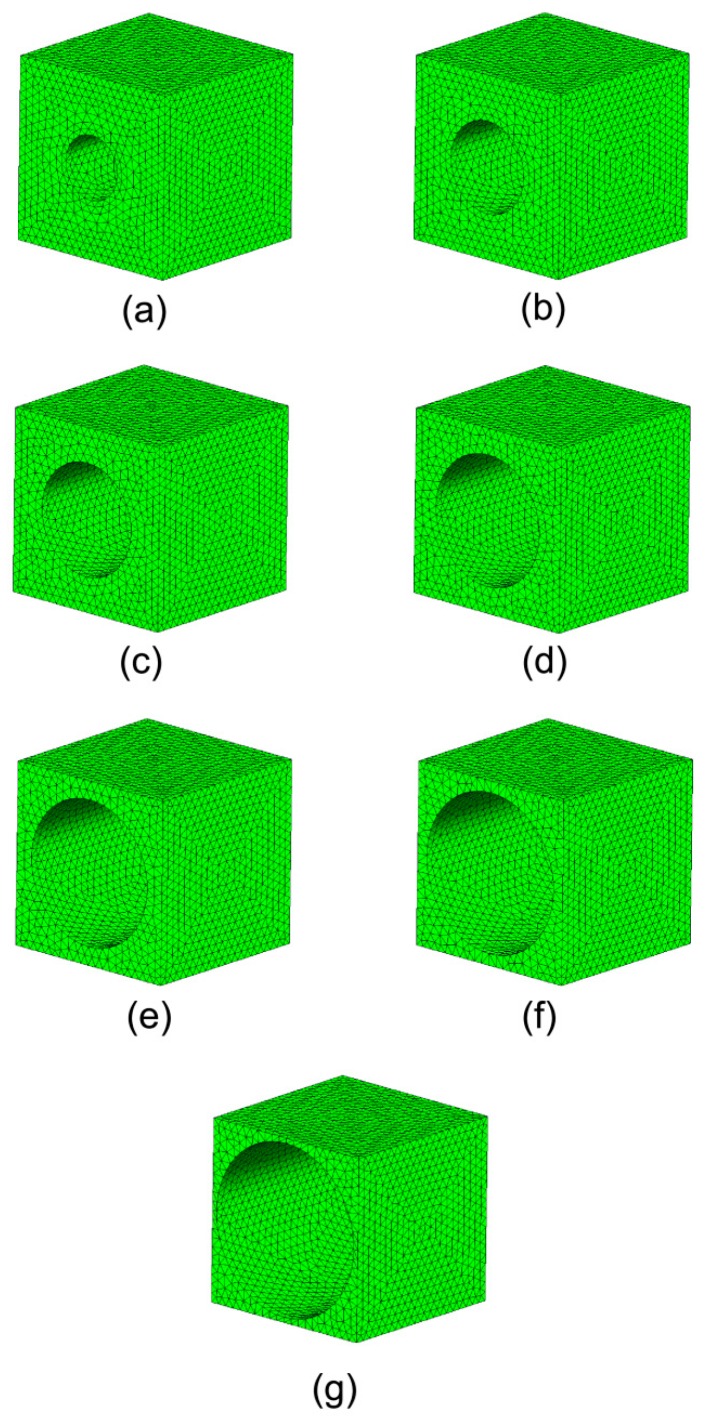
Mesh of solid cube with cylinder void inclusion for Darcy’s permeability homogenization. Void fractions (**a**) 0.1, (**b**) 0.2, (**c**) 0.3, (**d**) 0.4, (**e**) 0.5, (**f**) 0.6, (**g**) 0.7.

We considered different void fractions as shown in Figure 11. This problem was studied by Gebart [82], giving an analytical formula both for the longitudinal (along the fiber/cylinder direction) and transverse permeability, namely,

$$\begin{array}{c} k_{L,G}=\frac{8}{57}\frac{{\left(1-V_{f}\right)}^{3}}{V_{f}^{2}}r^{2}\\ k_{T,G}=\frac{16}{9\pi \sqrt{2}}{\left(\sqrt{\frac{\pi }{4V_{f}}}-1\right)}^{2.5}r^{2} \end{array}$$

with $V_{f}$ the volume fraction and r the radius of the cylinders. Berdichevsky and Cai [83] on the other hand presented the following formulae for the permeabilities of the same problem,

$$\begin{array}{l} k_{L,B}=\frac{r^{2}}{8V_{f}}\left(\ln\frac{1}{V_{f}^{2}}-\left(3-V_{f}\right)\left(1-V_{f}\right)\right)\\ k_{T,B}=\frac{r^{2}}{8V_{f}}\left(\ln\frac{1}{V_{f}^{2}}-\frac{{\left(1-V_{f}\right)}^{2}}{{\left(1+V_{f}\right)}^{2}}\right) \end{array}$$

**Figure 12:**
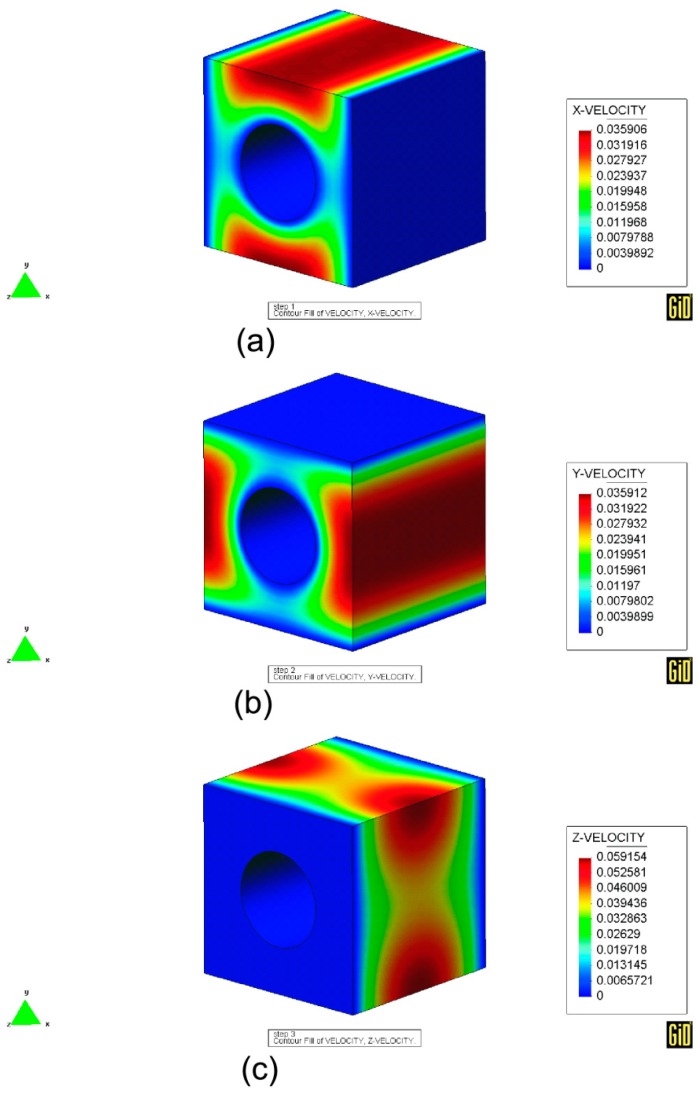
Characteristic velocity field of solid cube with cylinder void inclusion (0.2). (**a**) $\kappa_{1}^{1}$, (**b**) $\kappa_{2}^{2}$, (**c**) $\kappa_{3}^{3}$.

**Figure 13:**
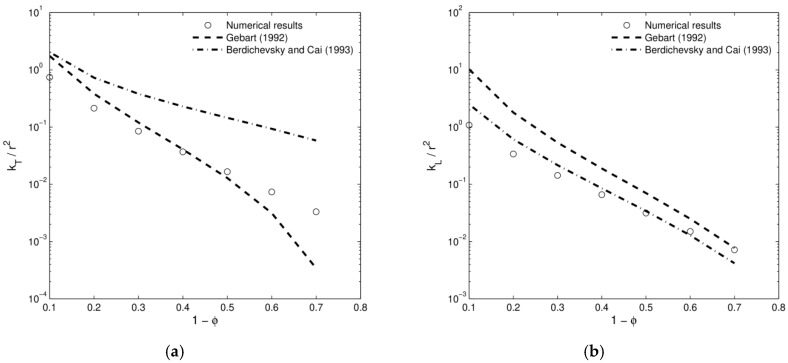
Theoretical estimates [82,83] versus numerical values of dimensionless permeability of a body with a cylinder void (radius r, void fraction ϕ). (**a**) transverse permeability; (**b**) longitudinal permeability.

The solution of the so-called characteristic velocities is given in Figure 12. The remaining functions $\kappa_{i}^{j}$, which are not presented in that figure, are negligible. Finally, a comparison of the obtained values and the theoretical estimates are shown in Figure 13. Numerical results are always comparable showing a better fitting with the solution provided by [82] for the transverse permeability and [83] for the longitudinal one.

This technique has been successfully applied for the determination of the permeability tensor, and hence the macroscopic Darcy’s law, in woven structures [84,85], tissue engineering scaffolds [86,87,88] and porous materials [70,89].

### 2.4. Heat Transfer

In another field of application, heterogeneous materials with microstructure look for having excellent heat conduction/isolation macroscopic properties. Some examples are isolating construction materials (building materials, mechanical parts of engines), heat exchangers or thermal energy storage materials. All these problems may be analyzed by means of the heat transfer (Fourier) law both macroscopically and microscopically (note that the presented analysis here is also available for Fickean diffusion due to the analogy of the mathematical equations. The difference of this approach from that presented in Section 2.3 is here the microstructural level is a heterogeneous material composed of different phases with different diffusion coefficients. The pore scale which drives the diffusion mechanism is not then captured yet microscopically as it is in the approach presented in Section 2.3.). Therefore, macroscopic heat transfer behavior is given by analyzing the microscopic heat transfer problem (Note that the microscopic problem is still formulated in phenomenological terms).

Heat transfer equation in the steady state at the micro level can be written as

(33) $$\begin{array}{c} \nabla \cdot q=0\\ q=-D\cdot \nabla \theta \\ \langle q\rangle =q^{M} \end{array}$$

q being the heat flux at the micro-level, D the heat conductivity matrix and θ the temperature. Having in mind the asymptotic decomposition of the temperature field $\theta \left(X,Y\right)=\theta^{M}\left(X\right)+\theta^{m}\left(Y\right)$, one can identify the steady-state heat transfer problem as the scalar version of the multiscale Poisson problem in linear elasticity presented in Section 2.1. Therefore, the mathematical details of the problem in this section are avoided due to that analogy.

The homogenization technique has been used in [90,91] for the analytical estimation of thermal properties. More recently, [92,93,94,95] have derived a multiscale formulation of heat transfer problems.

Specifically, heat transfer problems have many interests in composites sciences since these structures are usually subjected to extreme temperature changes. It is important then to predict apparent thermal conductivities in order to analyze the mechanical behavior and the degradation process in composite structures. This has been studied analytically in [96,97,98], by the asymptotic homogenization method [99] or following a multiscale procedure [100,101].

### 2.5. Multiphysics: Thermomechanics, Poroelasticity, and Others

The multiscale treatment of several multiphysics problems of interest is reviewed in this section.

#### 2.5.1. Thermomechanics

Thermomechanical analysis of heterogeneous materials is of interest in applications such as ceramic coatings, high temperature alloy pipes, MMC in combustion engines, turbines, etc. These problems involve the coupling of the mechanical and heat transfer problems, so the multiscale formulation of the thermomechanical problem is based on the natural extension of the linear elasticity and heat transfer problems. Therefore, the thermomechanical macroscopic behavior is given by analyzing the microscopic thermomechanical problem (as in Section 2.4 the microscopic problem is still formulated in phenomenological terms).

Micromechanically, the linear thermomechanical problem is written as follows,

(34) $$\begin{array}{c} \nabla \cdot \sigma -\rho \ddot{u}=0\\ \varepsilon =\frac{1}{2}\left(\nabla u+{\left(\nabla u\right)}^{T}\right)\\ \sigma ={\mathbb{C}}^{m}:\varepsilon +V\dot{\varepsilon }-\mathbb{C}:\alpha \theta \\ \rho c_{p}\dot{\theta }=\nabla \cdot q+Q+V\dot{\varepsilon }⊗\dot{\varepsilon }\\ q=-D\cdot \nabla \theta \\ \langle \varepsilon \rangle =\varepsilon^{M}\\ \langle q\rangle =q^{M}\\ +\;\mathrm{initial}\;\mathrm{and}\;\mathrm{boundary}\;\mathrm{conditions} \end{array}$$

with ρ the density, V the viscosity tensor (considering a Kelvin–Voigt model), α the coefficient of thermal expansion vector, $c_{p}$ the specific heat per unit mass, Q the volumetric heat source term and $V\dot{\varepsilon }⊗\dot{\varepsilon }$ the mechanical (viscous) dissipation.

Neglecting (in other words, we mean that the time needed to impose changes to the mechanical and thermal loading at the macroscale is assumed to be much larger than the time required for the velocity and temperature of the microscopic problem to reach the steady state due to such changes [102]) inertial forces at the microscale since the volume of the RVE is small [27] and also thermal inertial effects at the microscale, i.e., first left term in Equation (34); and considering the asymptotic decomposition of both the displacement and temperature field, namely, $u\left(x,Y\right)=u^{M}\left(X\right)+u^{m}\left(Y\right)$ and $\theta \left(x,Y\right)=\theta^{M}\left(X\right)+\theta^{m}\left(Y\right)$, respectively, the localization and homogenization associated problems to (34) can be formulated by analogy to the concepts of localization and homogenization of linear viscoelasticity and heat transfer problem. Heat transfer has been analyzed in Section 2.4. On the other hand, localization and homogenization in linear viscoelasticity can be developed by turning the viscoelastic problem into a fictitious elastic one by means of the Laplace transform. Then, the process is analogous to the linear elasticity presented in Section 2.1. The bases of the localization and homogenization of the linear viscoelastic problem are given in [41]. Nonetheless, localization and homogenization problem setup for coupled thermomechanics, i.e., Equation (34), is shown in [103]. A modern multiscale approach to this problem is found in [104].

**Figure 14:**
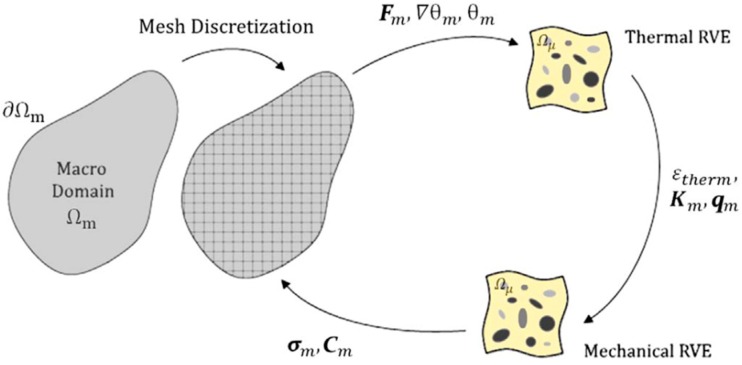
Multiscale thermomechanical solving scheme (taken with permission from [105]). The heterogeneous microscopic structure is evaluated stepwise and upscaled to the macroscopic problem in order to assess the long-term performance of the tubes.

An interesting application of multiscale thermomechanical solvers in the optimization of solar selective coatings for absorber tubes can be found in [105]. In this application, the absorber layer is a nanocomposite consisting of an amorphous carbon matrix reinforced with titanium carbide nanoparticles. Therefore, RVEs containing one and several particles, respectively, were defined in order to obtain homogenized thermo-mechanical properties upscaled to the component level for the long-term performance analysis of the whole absorber tube. For this purpose, the strain and thermal gradients of the macroscopic problem are set as boundary conditions at the microscale boundary value problem. In the first stage, the heat transfer problem is solved and, then, the microscopic thermal strain field is used in the subsequent mechanical boundary value problem. Finally, both the microscopic stress tensor and heat flux vector are homogenized and upscaled to the macro problem, as represented in Figure 14. 

**Figure 15:**
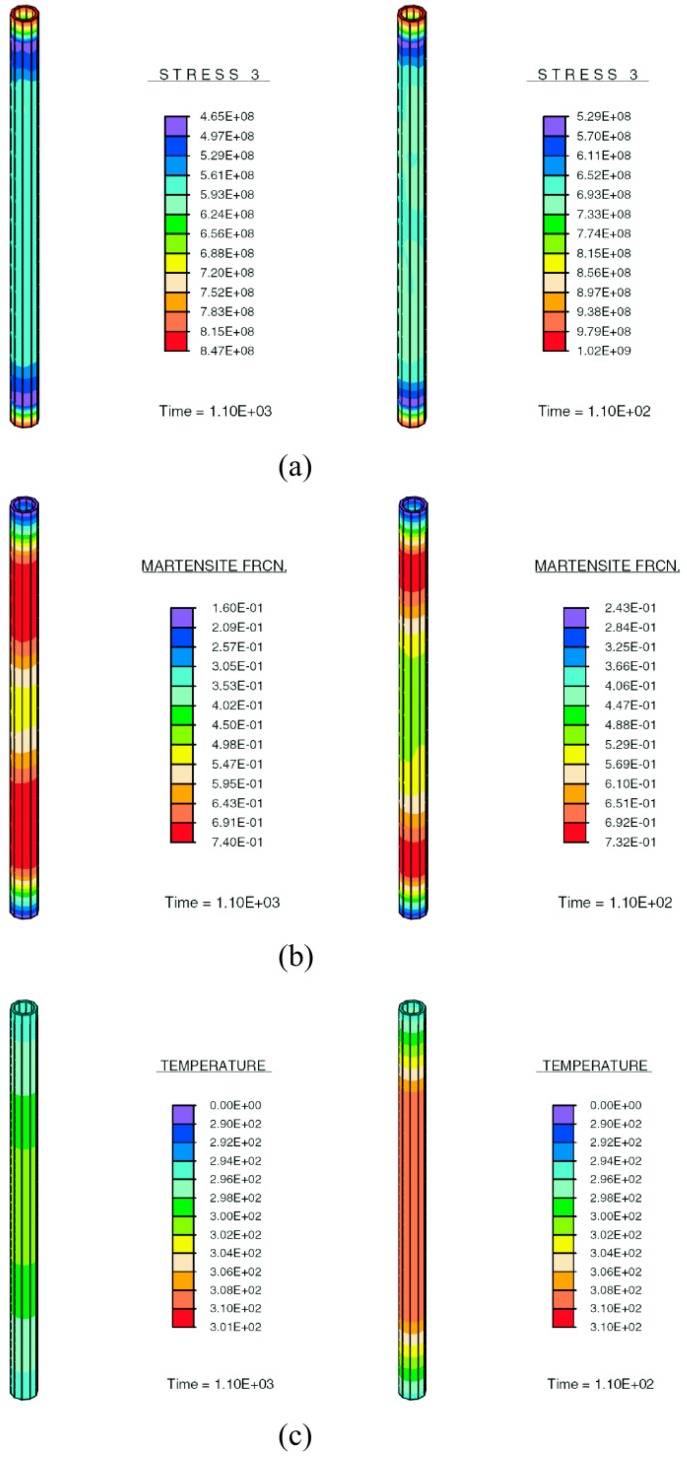
Macroscopic results of Nitinol tube under stretch (taken with permission from [102]). (**a**) Longitudinal Cauchy’s stress (Pa); (**b**) martensite fraction; and (**c**) temperature [K]. Left and right results for an imposed strain rate of 10^−4^ s^−1^ and 10^−3^ s^−1^, respectively.

Another interesting example is that of [102], in which a fully-coupled thermomechanics monolithic scheme is adopted to solve the momentum and energy equations and applied to shape-memory alloys (Figure 15).

#### 2.5.2. Poroelasticity

Poroelastic saturated media—such as soils, rocks, foams or living tissues—are microstructurally biphasic media composed by a solid skeleton and a fluid phase. Poroelasticity Biot’s macroscopic equations are then the phenomenological description of the microscopic fluid motion according to the microscopic deformation of the solid skeleton. Poroelastic macroscopic equations read as,

(35) $$\begin{array}{c} \sigma^{M}={\mathbb{C}}^{M}:\varepsilon_{S}^{M}-\beta p^{M}\\ \varepsilon_{S}^{M}=\frac{1}{2}\left(\nabla u^{M}+{\left(\nabla u^{M}\right)}^{T}\right)\\ \nabla \cdot \sigma^{M}=\rho {\ddot{u}}^{M}+\phi \rho_{f}{\dot{w}}^{M}\\ {\dot{\varsigma }}^{M}+\nabla \cdot g^{M}=0\\ g^{M}=-K\left(\nabla p^{M}+\rho_{f}{\ddot{u}}^{M}+\frac{\rho_{f}+\phi \rho_{f}}{\phi }{\dot{w}}^{M}\right)\\ +\;\mathrm{initial}\;\mathrm{and}\;\mathrm{boundary}\;\mathrm{conditions} \end{array}$$

The first line in Equation (35) relates the constitutive macroscopic relationship in terms of the macroscopic solid skeleton deformation $\varepsilon_{S}^{M}$ and the overall pore pressure $p^{M}$. In this equation, β is the Biot’s stress tensor defined as,

$$\beta =\phi \left(1+R^{-1}Q\right)$$

R and Q being the macroscopic tensors accounting for fluid-solid interactions, which are only dependent on the microarchitectural distribution and shape of pores. The second line in Equation (35) is the compatibility deformation condition for the solid skeleton, in the third one the dynamic equilibrium equation written in terms of the rule of mixtures ($\rho_{f}$ being the fluid density in that equation). The fourth line in Equation (35) is the mass balance equation, with $\dot{\varsigma }$ defined as the variation of fluid volume per volume. Finally, Darcy’s macroscopic law is given in, with $g^{M}$ the macroscopic specific flux.

Under the hypothesis of small strains, both the apparent elastic, fluid, and solid-fluid macroscopic properties in Equation (35) can be derived by a microstructural homogenization analysis analogous to those presented in Section 2.1 and Section 2.3 (see [70]). Moreover, the stress state at the microscopic solid skeleton as well as velocity profiles within the pore phase may be obtained by its associated microscopic localization problem (at the microstructural level, we neglect inertial forces analogously to the problem presented in Section 2.5.1). Note that, at the microscopic scale, a more fundamental physics—i.e., mechanics, fluids, and their interaction—may be distinguished as difference of the heat transfer or thermomechanical problems. 

The approach here followed can analyze the poroelastic features and homogenized macroscopic properties of porous media [106,107], tissue engineering scaffolds [108,109], or to derive a multiscale FE^2^ approach for poroelastic macroscopic media including plasticity [110].

#### 2.5.3. Others

An interesting multiscale and multiphysics problem can be identified in bone tissue regeneration using scaffolds, i.e., bone tissue engineering processes. As mentioned in the introduction, structural porous supports are set in the bone defect to promote new bone tissue regeneration. Microscopically, bone cells attached to the scaffold pore surface and segregate matrix and hence new bone tissue. Cell matrix generation is highly influenced on mechanical cues that ‘feel’ bone cells from the micromechanical state in their neighborhood and the fluid microcirculation within the pores. Overall, microscopic new tissue regeneration changes the apparent elastic and permeability scaffold properties. Macroscopically, the inclusion of a scaffold in the bone defect induces a different mechanical state in the surrounding of the implant, which activates a remodeling process in bone.

**Figure 16:**
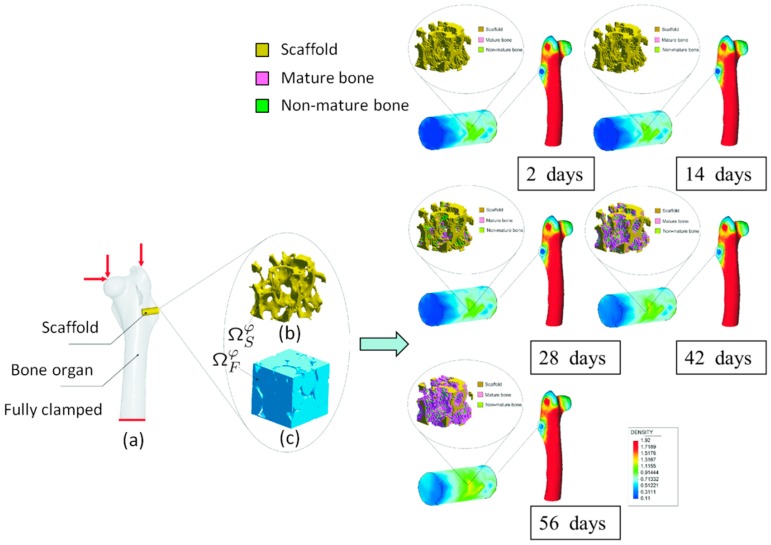
Multiscale and multiphysics bone tissue regeneration using biodegradable scaffolds. (**a**) Proximal femur implanted with a scaffold in the greater trochanter region. A detail of the microstructure is shown in (**b**) solid domain and (**c**) fluid domain. Right: Apparent (macroscopic) density evolution (g cm^−3^) of the bone organ and detail of the scaffold implantation. Microscopically, bone regeneration distribution onto the scaffold microsurface of the macroscopic scaffold midpoint is shown for different days after implantation. See [24].

This problem was approached in [24] using a multiscale and multiphysics approach (see Figure 16). The macroscopic domain was divided into bone and scaffold regions. In the bone domain, the linear elasticity equations with a certain macroscopic bone remodeling model were solved. The scaffold domain was treated as a multiscale and multiphysics approach. In this domain, fluid and solid skeleton domains were distinguished (see Figure 16). New bone tissue deposition, scaffold degradation as consequence of biodegradation as well as the mechanical behavior of the solid skeleton were modeled at this two-scale level. The apparent mechanical and permeability properties were homogenized at the macroscale taking into consideration the evolution of the microstructure as consequence of tissue regeneration and scaffold resorption. Using this approach, the overall new bone tissue regeneration can be predicted in terms of the microscopic biophysics and mechanobiology that take place. The multiscale problem was formulated using the homogenization theory applied to solids and fluids introduced in Section 2.1 and Section 2.3 (see [24]). Figure 16 shows the evolution of new bone tissue growth both macroscopically (in terms of density) as well as microscopically in the middle location of the implant for different days. This problem is globally nonlinear so that it gives rise to a FE^2^ solution strategy. The source of this nonlinearity is the evolution (change) with time of the different phases of the microstructure, due to the described phenomena.

## 3. Non-Homogenization-Based Multiscale Approaches

In some multiscale problems, the separation of scales, as introduced in Section 2, does not hold because $\ell_{m}\sim \ell_{M}$. Under such condition, the authors propose the term non-homogenization-based multiscale (NHM) approaches to encompass all those techniques that cannot be classified as HM.

NHM becomes the main alternative on the limits of pure HM methods. For instance, the concept of RVE, on which the idea of HM methods is based, does not work when material instabilities and localization are present (e.g., local buckling, cracks). The very alternative is to tackle the problem directly at the microscale. However, this may become computationally inefficient and, of course, tremendously expensive. These, often called true multi-scale methods can be globally summarized in the following list:

- Multigrid [111]
- Domain decomposition-based [112], embedded or static condensation [113]
- Wavelet methods [114]
- Discrete-to-continuum, also often referred to as mesoscale models (e.g., coarse-graining [67,115], quasi-continuum [116], bridging domain methods [30,117]).

There exists a plethora of multiscale techniques currently available. It is not the purpose of the authors to detail all of them but to provide a brief overview on the main ingredients of the formulation of these solvers. Certainly, the authors will focus on the first two approaches, namely, the multigrid and domain decomposition methods under different applications from non-linear mechanics to transport or heat transfer problems. The reader is referred to the literature [26,118,119] in order to get more details on other approaches, yet not restricted to continuum physics.

### 3.1. Multigrid

**Figure 17:**
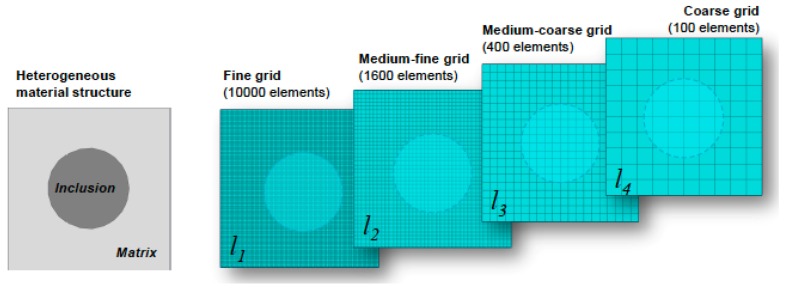
The multigrid method can be implemented for solving multiscale problems in heterogeneous materials. The heterogeneities are mapped onto the different grids (from the finest to the coarsest).

Multigrid methods are iterative solvers that exchange information on the solution among different grids (e.g., discretizations) of the same problem by means of transfer operators and local processing at each scale. The basis of multigrid methods can be found in [111] in which it is defined as an algebraic method for solving discrete equations (e.g., finite differences, finite elements) on a given grid by constant interaction with a hierarchy of coarser grids (as shown in Figure 17). In fact, that multigrid approach is based on a multilevel hierarchy that it makes the methodology applicable to any numerical method for solving the continuum (if these are based on the discretization of the domain).

The most general approach in multigrid is the so-called V-cycle by which a first stage of coarsening is followed by the determination of the exact solution and then the interpolation and relaxation.

Given a positive definite and symmetric matrix, K, and the linear system, Ku=f, the energy minimization problem reads

(36) $$P\left(u\right)=\frac{1}{2}u^{T}Ku-u^{T}f$$

Now, define $\bar{u}$ as the current approximation of the solution; then, the absolute error is defined as

(37) $$e=u-\bar{u}$$

The residual, therefore, can be defined as

(38) $$r=f-K\bar{u}=K\left(u-\bar{u}\right)=Ke$$

Then, at all levels, the problem is reduced to solving Ke=r. The minimization problem then reads as

(39) $$\min\left\{\frac{1}{2}e^{T}Ke-e^{T}r\right\}=\min\left\{\frac{1}{2}{\left(\tilde{e}+H_{c}^{f}e^{c}\right)}^{T}K\left(\tilde{e}+H_{c}^{f}e^{c}\right)-{\left(\tilde{e}+H_{c}^{f}e^{c}\right)}^{T}r\right\}$$

where $\tilde{e}$ is the initial fine level error, $e^{c}$ the coarse level error, and $H_{c}^{f}$ the coarse-to-fine interpolation operator. 

The multigrid method, according to Brandt [111], has a double-side view depending on the coarsening or refining of the grids. For instance, in the case of coarse grids, it can be seen as “correction grids accelerating convergence of a relaxation scheme on the finest grid by efficiently liquidating smooth error components. On the other hand, finer grids can be seen as connecting grids that improve accuracy on coarser grids by connecting their forcing terms. So, it is possible to manipulate accurate solutions on coarser grid with infrequent visits to pieces of finer levels”.

#### 3.1.1. Non-Linear Mechanics

In [26], the Newton-multigrid algorithm is presented for the resolution of multiscale non-linear mechanics problems focusing in large plastic strains. The solution of the non-linear problem is based on the linearization of the system

(40) $$\mathrm{Lin}\left\{r\left(\Delta u\right),u_{0}\right\}=r\left(u_{0}\right)+K\left(u_{0}\right)\Delta u=0$$

with $K\left(u_{0}\right)=\frac{\partial r\left(u_{0}\right)}{\partial u}$.

**Figure 18:**
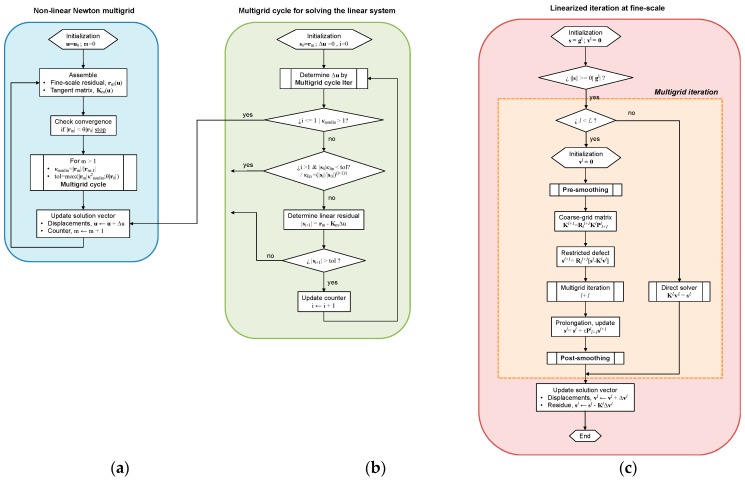
Multigrid schemes: non-linear Newton multigrid (**a**) calls a nested algorithm for solving the linear problem (**b**) and, at the same time, this calls for a linearized iteration at the fine-scale (**c**). Algorithm adapted from [26].

The algorithm for the non-linear mechanics problem is presented in Figure 18 adapted from [26]. The Newton-type solver for the non-linear problem (Figure 18a) calls an iterative multigrid cycle (described in Figure 18b) for the linearized problem.

This scheme provides an important speed-up with respect to direct solvers. According to the authors, the critical point with regard the efficiency of such a method is the consideration of the heterogeneity of the linearized structural problem. Finally, in order to reduce the fine-scale oscillating part, a two-grid cycle with pre- and post-smoothing is used to solve the problem at the fine-scale.

The main transfer functions are defined in this method in order to promote the prolongation, i.e., coarse-to-fine transformation, $P_{l+1}^{l}$; and the restriction, i.e., the fine-to-coarse transformation, $R_{l}^{l+1}$. For symmetric matrices, $R_{l}^{l+1}={\left(P_{l+1}^{l}\right)}^{T}$.

In the aforementioned work, the heterogeneity of the microstructure is tackled by splitting the fine-scale displacements, v, into a long-wave and a short-wave term

(41) $$v=\bar{v}+\tilde{v}$$

Similarly, the transfer operators can be split into these two types of fluctuations; therefore

(42) $$\begin{array}{c} P_{c}^{f}={\bar{P}}_{c}^{f}+{\tilde{P}}_{c}^{f}\\ R_{f}^{c}={\bar{R}}_{f}^{c}+{\tilde{R}}_{f}^{c} \end{array}$$

where c and f stand for coarse and fine, respectively.

#### 3.1.2. Darcy Flow

Geomechanical information is usually provided at very high resolution; therefore, it is difficult to handle this type of problems at the full-scale. In this sense, coarsening of the information is very interesting but due to the multiscale nature in the physics of the problem, it cannot be treated as an isolated problem. Multigrid methods provide a useful framework for tackling this type of problems. Indeed, heterogeneity in the permeability can be treated at the finer scale and the solution can be approximated at the coarser scale [120].

The fluid flow through a porous medium is described by Darcy’s law, that reads

(43) $$\bar{u}=-\frac{\kappa }{\mu }\left(\nabla p-\rho g\right)$$

where $\bar{u}$ is the macroscopic velocity field in the porous domain, p the pressure field, g the gravity field, κ the permeability, μ the viscosity, and ρ the density.

The continuity equation is

(44) $$\nabla \cdot \bar{u}=f$$

f being the source term. The problem is completely defined with the boundary conditions in terms of flow and pressure

(45) $$u\cdot n=g_{N}$$

(46) $$p=g_{D}$$

where n is the normal outer vector of the boundary and $g_{N}$ and $g_{D}$ the Neumann’s and Dirichlet’s values for the boundary conditions, respectively.

The problem is reduced to finding u and p, which can be expressed in terms of a linear system of equations as

(47) $$\left[\begin{array}{cc} 0 & B^{T}\\ -B & D \end{array}\right]\left[\begin{array}{c} p\\ u \end{array}\right]=\left[\begin{array}{c} b\\ c \end{array}\right]$$

with $b=f-\nabla \cdot v_{g_{N}}$, $c=\rho g-kv_{g_{N}}$, B the differential operator, and D the inverse of the permeability tensor that, in the case of isotropic media, is defined as $D=\kappa \mu^{-1}I$, with I as the identity tensor. $v_{g_{N}}$ is the velocity on the Neumann’s boundary such that $v_{g_{N}}\cdot n=0$, with n the outwards normal vector. The velocity is defined as $\bar{u}=u+v_{g_{N}}$.

The multigrid algorithm for Darcy’s flow presented in [120] is based on the Schur complement of the velocities, which reads

(48) $$Ap=\left(B^{T}D^{-1}B\right)p=f=b-B^{T}D^{-1}c$$

**Figure 19:**
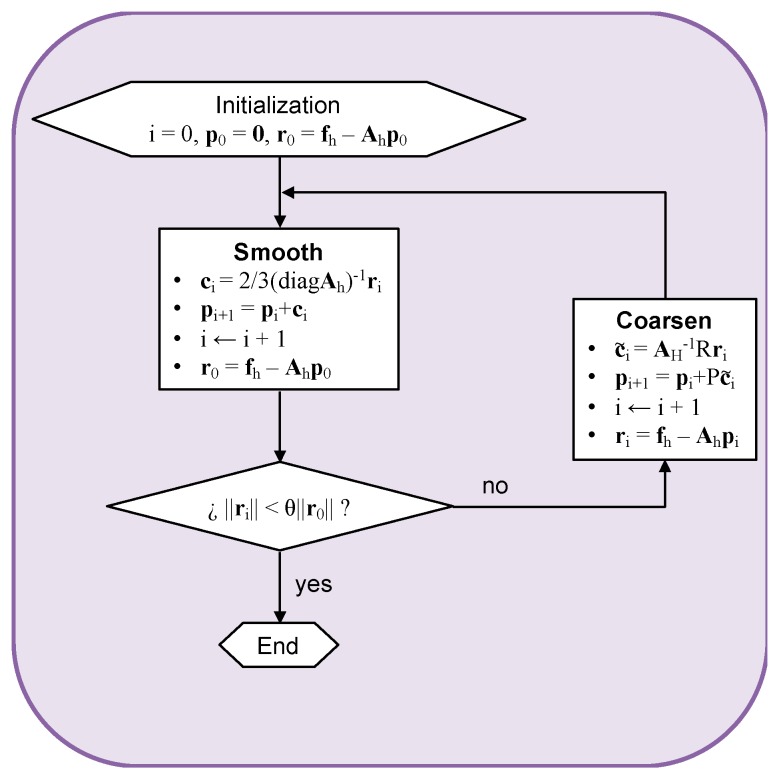
Multigrid scheme adapted from [120] for the Darcy problem. It consists of a two-level V-cycle with smoothing and coarsening of the solution.

In the work by Rath [120], a multigrid method is used to perform the coarsening procedure following an upscaling approach. The main objective is to achieve a compromise solution at the coarse scale sufficiently representative of the finer-scale. As shown in Figure 19, a two-level V-cycle is used to solve the linear system. The pressure is obtained using the residual, $r_{i}=f_{h}-A_{h}p_{i}$, where the subscript h refers to the fine-scale grid, and the coarse corrector $A_{H}$, where subscript H refers to the coarse-scale.

#### 3.1.3. Heat Transfer

Similarly, to the mechanical and the transport problem, heat transfer analysis is very challenging from the computational point of view. Moreover, when mechanical loads are present in such analysis (e.g., contact problems), the required computational power increases dramatically. Of course, when multiscale materials are being taken into account, alternative approaches to conventional direct simulations of the fine scale have to be foreseen.

In [121], a multigrid approach is proposed for the resolution of the heat transfer problem in the case of moving heat sources, as is the case of contact in surface coatings. The multigrid algorithm is based on a finite difference framework.

The differential equation of heat conduction for unsteady problems with no heat generation reads

(49) $$\rho c_{p}\frac{\partial \theta }{\partial t}-\nabla \cdot \left(D\nabla \theta \right)=0$$

with D the conductivity tensor, ρ the density, $c_{p}$ the specific heat, and θ the temperature field.

As mentioned before, the discretization framework in this work is the finite difference method, and the system can be linearized by defining a differential operator.

**Figure 20:**
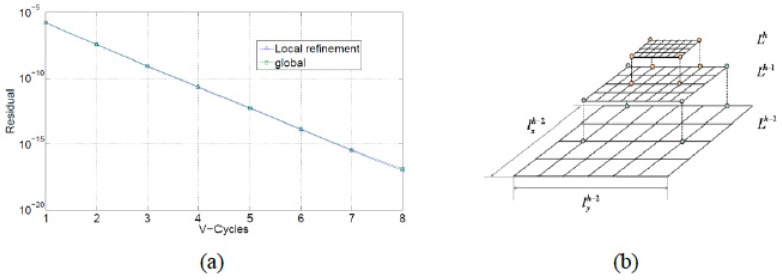
Residuals of the solution (**a**) for a unit flow disc heat transfer problem by means of a multigrid scheme (**b**). Taken with permission from [121].

According to Boffy et al. [121], multigrid methods are very convenient within local mesh refinement strategies: the fine grids are restricted to smaller subdomains, whereas the coarse grids cover the entire domain. The coarse grid correction is carried out in the local part, where the finest grid exists (see Figure 20).

In [122] more details on the implementation of the multigrid problem in V- and W-cycles are provided. The procedure is similar to that described in Section 3.1.1.

The approximation of the error is obtained on the coarse grid and interpolated to the target grid, this is used as a correction to improve the solution of this grid.

### 3.2. Domain Decomposition

It must be remarked that, in the case of domain decomposition, the definition of the displacement field differs from that of Section 2, since it is uniquely defined at each subdomain, as explained below. Thus, it makes no sense to define the displacement fields in terms of an average field plus the fluctuation term. For the sake of simplicity only one decomposition is considered in this section, but this could be extended to a general problem with a certain number of subdomains.

**Figure 21:**
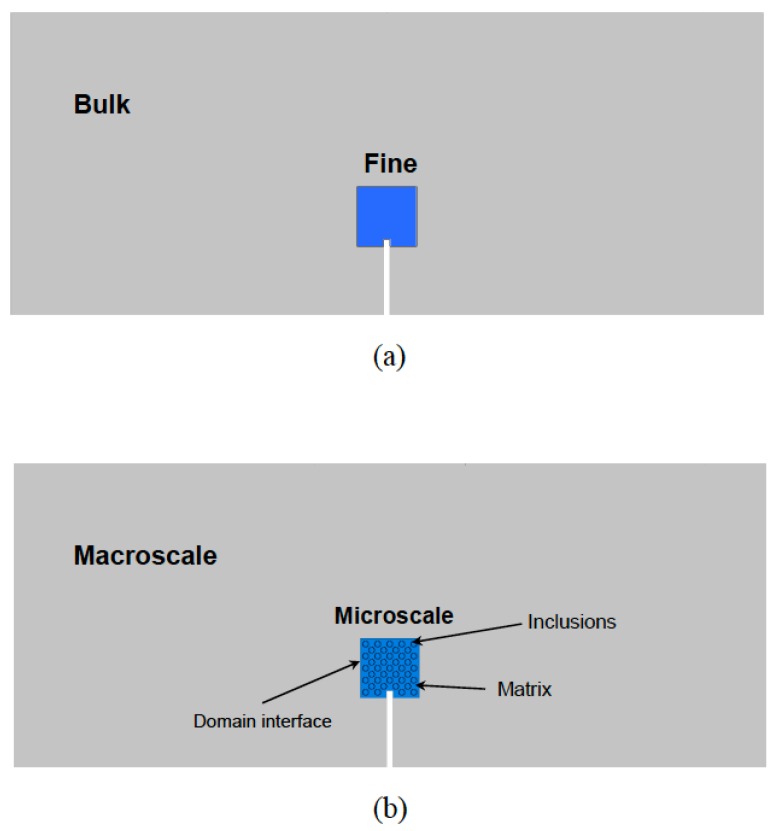
The domain decomposition method is a generic numerical technique that allows the interaction between different subdomains. For instance, it can be used to define one domain with bulk properties and another with homogenized properties (**a**). In the case of multiscale modeling, one subdomain corresponds to the macroscale model which interacts with the microscale model (matrix with cylindrical inclusions) through the interface (**b**). These figures correspond to a three-point bending test in notched specimens of a metal-matrix composite.

Let us consider the problem shown in Figure 21, where two zones within a global domain Ω are identified: one corresponding to the finer scale in which the microscale phenomena will be accounted for, $\Omega^{m}$; and the remainder material which is given bulk properties, $\Omega^{M}$. Thus, $\Omega \equiv \Omega^{m}\cup \Omega^{M}$. The interface between both subdomains is denoted as $\Gamma^{m-M}$.

The main idea in domain decomposition is that the problem has to be solved in each subdomain, $\Omega^{m}$ and $\Omega^{M}$, and continuity is enforced at the disjoint interface $\Gamma^{m-M}$. Another way of subdividing the domain is the so-called overlapping method, also known as Schwarz method [112].

#### 3.2.1. Linear Elasticity

As presented in Section 2, the linear elasticity problem for a given domain $\Omega^{k}$ and in the absence of body loads, can be written as

(50) $$\begin{array}{c} \nabla \cdot \sigma^{k}=0\\ \varepsilon^{k}=\frac{1}{2}\left(\nabla u^{k}+{\left(\nabla u^{k}\right)}^{T}\right)\\ \sigma^{k}={\mathbb{C}}^{k}:\varepsilon^{k} \end{array}$$

being $\sigma^{k}$ and $\varepsilon^{k}$ the stress and strain tensors, respectively, in the domain, and ${\mathbb{C}}^{k}$ the fourth-order linear elastic tensor containing the mechanical properties in the domain $\Omega^{k}$.

The problem above can be solved independently for each subdomain, k={m,M} with the following interface conditions:

- Dirichlet boundary conditions: $u^{m}=u^{M}$ on $\Gamma^{m-M}$
- Neumann boundary conditions: $\sigma^{m}\cdot n^{m}+\sigma^{M}\cdot n^{M}=0$ on $\Gamma^{m-M}$

Linear elastic domain decompositions methods are also useful for speeding up the analysis as they can be implemented in a general parallel framework, see for instance [123,124].

#### 3.2.2. Non-Linear Mechanics

In the case of nonlinearity in the material, the domain decomposition can be used to split the global domain into one linear problem (e.g., the bulk material—here defined as macroscale) and one non-linear problem (e.g., fine scale—here defined as microscale).

Thus, let us consider the linear elastic problem (Equation (50)) for the macroscopic domain (using superscript M), and the non-linear problem for the microscopic domain and defined in general as

(51) $$\begin{array}{c} \nabla \cdot \sigma^{m}=0\\ \varepsilon^{m}=\frac{1}{2}\left(\nabla u^{m}+{\left(\nabla u^{m}\right)}^{T}\right)\\ \sigma^{m}=f\left(\varepsilon^{m},{\dot{\varepsilon }}^{m},\;\Theta_{i}^{m},\ldots \right) \end{array}$$

As in the case of homogenization-based techniques, the stress tensor is evaluated in Equation (51) once defined its nonlinear constitutive relationship (e.g., plasticity, damage, etc.) which may eventually be a function of the strain and strain rate ($\varepsilon^{m}$ and ${\dot{\varepsilon }}^{m}$, respectively); and of internal variables denoted as $\Theta_{i}^{m}$.

In principle, there are not any special differences with respect to the liner elasticity approach, since the macro- and microscopic subdomains are solved independently, thus the interface conditions are kept:

- Dirichlet boundary conditions: $u^{m}=u^{M}$ on $\Gamma^{m-M}$
- Neumann boundary conditions: $\sigma^{m}\cdot n^{m}+\sigma^{M}\cdot n^{M}=0$ on $\Gamma^{m-M}$

#### 3.2.3. The Finite Element Tearing and Interconnecting (FETI) Method

Regarding a general concurrent scheme, one representing the microscale (m) where localization occurs and the remaining macroscopic material (M) which is assumed to undergo classical elastic behavior. For this type of models, a localization limiter may be required to determine whether a macroscopic zone has to be refined or not in an adaptive scheme. In [125], different coupling schemes (e.g., mortar and Arlequin methods) and a discussion on their advantages is driven.

The domain decomposition methodology presented by [126] for the multiscale analysis of heterogeneous brittle materials with continuum damage models is adapted for the continuum-discrete coupling. This approach is based on the finite element tearing and interconnecting (FETI) method. The microscopic model (in this case continuum) is used to analyze the fracture processes in the damaged zones, while the rest of the structure remains far from the inelastic regime and is analyzed with standard finite elements (see Figure 21).

Let us now consider the general variational formulation of the elasticity problem in a domain $\Omega \equiv \Omega^{m}\cup \Omega^{M}$,

(52) $$\int_{\Omega }\sigma :\dot{\varepsilon }d\Omega =\int_{\Omega }q\dot{u}d\Omega +\int_{\Gamma }T\dot{u}d\Gamma$$

where q are the body forces acting in Ω, T is the surface prescribed tractions on the boundary Γ such that T=σ⋅n on Γ, and $\dot{u}$ the virtual displacements rate.

The discretized version of (52), using a finite element approach, can be written as $f^{k}=K^{k}u^{k}$, where $u^{k}$ is the discrete approximation of the displacement field (i.e., nodal displacements) with k={m,M}. Then, the nodal forces vector $f^{k}$ and stiffness matrix $K^{k}$ are defined as

(53) $$\begin{array}{c} f^{k}=\int_{\Omega^{k}}Nq^{m}d\Omega +\int_{\Gamma^{k}}NT^{m}d\Gamma \\ K^{k}=\int_{\Omega^{k}}{\left(B^{k}\right)}^{T}D^{k}B^{k}d\Omega \end{array}$$

being $B^{k}$ the deformation matrices, N the shape functions derivatives matrix and $D^{k}$ the constitutive matrix such that $\sigma^{k}=D^{k}\varepsilon^{k}$. In the case of non-linear behavior, this matrix must be examined for each variable and computed as $D^{k}\approx \frac{\partial \sigma }{\partial \varepsilon }$. Thus, the matrix formulation of the fine and coarse scales reads, respectively, as

(54) $$\begin{array}{l} f^{m}=K^{m}u^{m}\;\mathrm{in}\;\Omega^{m}\\ f^{M}=K^{M}u^{M}\;\mathrm{in}\;\Omega^{M} \end{array}$$

Once the individual problems corresponding to each scale have been defined, we must enforce the continuity equation for their interface, $\Gamma^{m↔M}$:

(55) $$Q^{m}u^{m}+Q^{M}u^{M}=0$$

where $Q^{m}$ and $Q^{M}$ are the Boolean signed matrices, defined as

(56) $$Q^{m}=\{\begin{array}{c} 0\;\forall i,j\in \Omega^{m}\\ \pm 1\;\forall i,j\in \Gamma^{m↔M} \end{array}$$

for the fine scale domain, and equivalently for the coarse scale domain, $\Omega^{M}$.

The solution of Equation (54) with the restriction imposed in Equation (55) can be achieved by means of Lagrange multipliers. In fact, this method reduces a restricted problem with n variables to an unrestricted one with n+k variables, being k the number of restrictions.

#### 3.2.4. Darcy and Fick Problems

Domain decomposition methods have also been successfully applied in the field of Computational Geosciences, in order to describe the behavior in porous media flow [118,127]. For instance, one sound problem in the aforementioned topic is that of a fluid region, $\Omega^{f}$, that filtrates into a porous medium, $\Omega^{p}$. The former domain is governed by Stokes equations while the latter is ruled by Darcy equations. Thus, for the fluid domain, we have

(57) $$\begin{array}{c} -\nabla \cdot T\left(u^{f},p^{f}\right)=f\\ \nabla u^{f}=0\\ T\left(u^{f},p^{f}\right)=2\nu D\left(u^{f}\right)-p^{f}I\\ D\left(u^{f}\right)=\frac{1}{2}\left(\nabla u^{f}+{\left(\nabla u^{f}\right)}^{T}\right) \end{array}$$

where $u^{f}$ and $p^{f}$ are the displacement and pressure fields, respectively, in the fluid domain, T is the stress tensor, and D the deformation tensor. Equation (57) applies in the domain $\Omega^{f}$. On the other hand, the Darcy equation reads as

(58) $$\begin{array}{c} u^{p}=-\frac{1}{n}K\nabla p^{p}\\ \nabla u^{p}=0 \end{array}$$

where $u^{p}$ is the displacement field in the porous domain, $\Omega^{p}$, n the volumetric porosity, K the permeability tensor, and $p_{p}$ the pressure gradient. Equation (58) applies for the domain $\Omega^{p}$.

Similarly, to the mechanical problem, both domains interact at the interface, here defined as $\Gamma^{p↔f}$, with the following conditions:

- Conservation of mass: $u^{f}\cdot n^{f}=u^{p}\cdot n^{p}$ on $\Gamma^{p↔f}$
- Balance of normal forces: $p^{f}-2\nu {\left(n^{f}\right)}^{T}D\left(u^{f}\right)n^{f}=p^{p}$ on $\Gamma^{p↔f}$
- Beavers-Joseph-Saffman condition: $u^{f}\cdot T^{f}=\zeta {\left(s^{p↔f}\right)}^{T}D\left(u^{f}\right)s^{p↔f}$ on $\Gamma^{p↔f}$

where $n^{f}$ and $n^{p}$ are normal unit vectors defined outwards the corresponding domain, $s^{p↔f}$ the tangential unit vector of the interface $\Gamma^{p↔f}$, and ζ a representation of the characteristic length of the pores.

In [128], different iterative methods are presented in order to compute the solution of the coupled problem by solving independently the fluid and porous problems and imposing Robin conditions contrary to using Neumann–Dirichlet methods which are strongly sensitive to the fluid viscosity and permeability tensor. In the referred paper, complete details on the numerical implementation of the weak form of the problem can be found.

One interesting application of the domain decomposition method to diffusion equations is that of neutron flux. In [129], a methodology for analyzing the neutron diffusion problem following a non-overlapping Schwarz decomposition with Robin interface conditions. The diffusion problem reads

(59) $$\begin{array}{c} \frac{1}{D}p+\nabla \phi =0\\ \nabla \cdot p+S_{a}\phi =Q_{g} \end{array}$$

being p the neutron current and ϕ the neutron flux, D the diffusion coefficient, $S_{a}$ the absorption cross section, and $Q_{g}$ the neutronic source representing secondary neutrons from scattering and fission reactions.

Now assume two domains with different scales of resolution, namely one for the macroscale, $\Omega^{M}$, and another for the microscale, $\Omega^{m}$. Thus, the neutron current and flux are defined in both domains, $p^{M}$ and $\phi^{M}$ for the macroscale; and $p^{m}$ and $\phi^{m}$ for the microscale. The interface conditions applied on $\Gamma^{m↔M}$ are:

- Flux continuity: $\phi^{m}=\phi^{M}$ on $\Gamma^{m↔M}$
- Balance of neutron current: $p^{m}\cdot n^{m}+p^{M}\cdot n^{M}=0$ on $\Gamma^{m↔M}$

with $n^{m}$ and $n^{M}$ the normal unit vectors on the interface, defined outwards. In [129], Robin interface conditions are used instead, thus

(60) $$\begin{array}{l} p^{m}\cdot n^{m}+\alpha^{m}\phi^{m}+p^{M}\cdot n^{M}+\alpha^{m}\phi^{M}=0\\ p^{m}\cdot n^{m}+\alpha^{M}\phi^{m}+p^{M}\cdot n^{M}+\alpha^{M}\phi^{M}=0 \end{array}$$

where $\alpha^{m},\alpha^{M}>0$.

#### 3.2.5. Heat Transfer

Let us consider the conduction steady-state heat transfer problem with two domains. The macroscopic domain, $\Omega^{M}$, is characterized by its conductivity tensor $K^{M}$. However, a microscopic domain, $\Omega^{m}$, with a higher resolution conductivity tensor $K^{m}$ may be required. The steady-state heat transfer equation for the macroscopic domain reads as

(61) $$-\nabla \cdot \left(K^{M}\nabla T^{M}\right)=Q^{M}$$

being $T^{M}$ the temperature field and $Q^{M}$ the heat source term which is assumed null below. Similarly, the heat transfer problem at the microscale reads

(62) $$-\nabla \cdot \left(K^{m}\nabla T^{m}\right)=Q^{m}$$

and no volumetric heat source will be considered next, $Q^{m}=0$.

Within a general domain decomposition scheme [130], the interface conditions that must be enforced on $\Gamma^{m↔M}$ are

- Flux continuity: $q^{m}\cdot n^{m}+q^{M}\cdot n^{M}=0$ on $\Gamma^{m↔M}$
- Temperature continuity: $T^{m}=T^{M}$ on $\Gamma^{m↔M}$

where $n^{m}$ and $n^{M}$ are the normal unit vectors at the interface $\Gamma^{m↔M}$ in the micro- and macroscale domain, respectively, $q^{m}$ and $q^{M}$ are the heat fluxes defined as

(63) $$\begin{array}{l} q^{m}=-K^{m}\nabla T^{m}\\ q^{M}=-K^{M}\nabla T^{M} \end{array}$$

#### 3.2.6. Illustrative Example

In order to remark the main features of the domain decomposition technique, an illustrative example is presented below. Let us consider the three-point bending test (3PBT) of a metal-matrix composite notched specimen. The microstructure has been idealized a regular triangular arrangement of circular inclusions of a softer material. The matrix is assumed to behave as an elastic perfectly-plastic material, while the inclusions are pure elastic.

**Figure 22:**
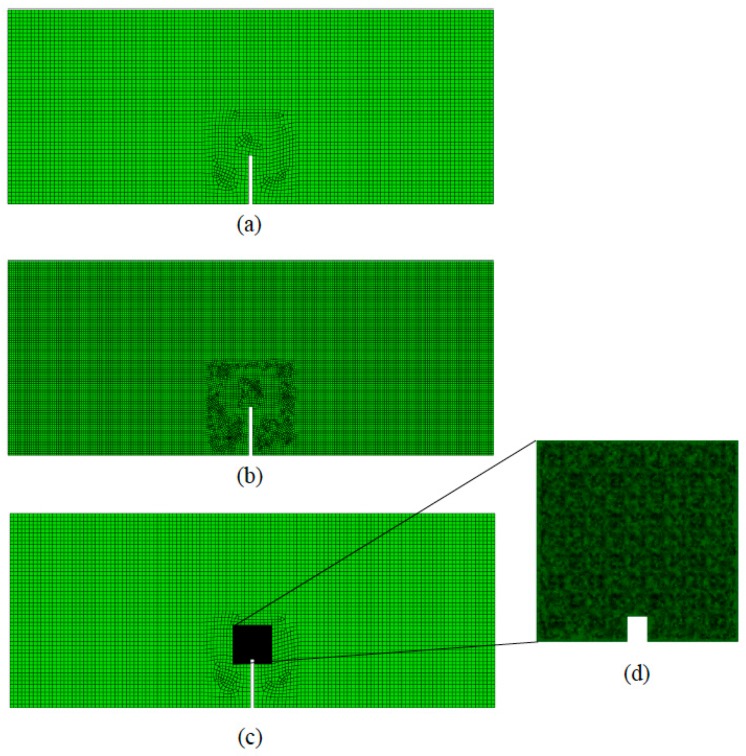
Domain decomposition should not be mistaken with mesh refinement. Figure (**a**,**b**) show a macroscale model with coarse and fine meshes, while a multiscale domain with macroscale (**c**) and microscale (**d**) subdomains may have different mesh density.

In this example, two approaches are presented: the first one consists in using a homogenized elastic-plastic response for the whole domain (Figure 22a,b), ($\Omega^{m}\cup \Omega^{M}$); while in the second one the domain is decomposed into a macro- and microscopic subdomain (Figure 22c,d). In the former approach, the actual material heterogeneity is not taken into account and two different meshes, coarse and fine, were used; while in the latter approach the heterogeneity is included in the microscopic domain and the mesh size for the macroscopic subdomain is that of the fine homogenized case (Figure 22b) and a finer mesh for the microscopic subdomain in order to model the different phases.

**Figure 23:**
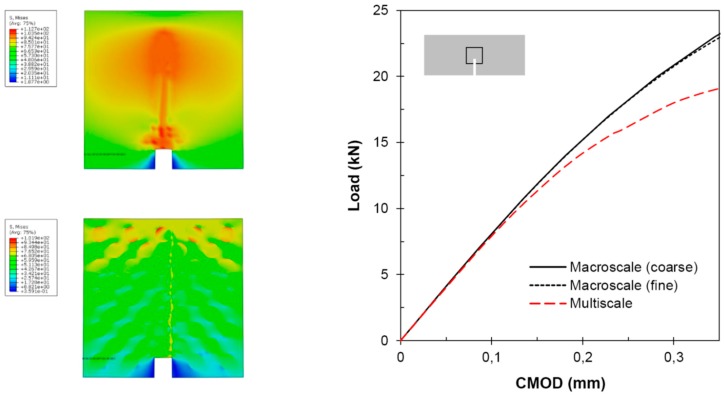
The presence of heterogeneities at the microscale is determinant on the macroscopic response. In fact, two mesh densities provide a similar load-crack mouth opening displacement (CMOD) response which overestimate that of a multiscale model accounting for heterogeneities at the microscale. This observation becomes more evident in the stress field distribution (units in MPa).

In Figure 23, the load versus crack mouth opening displacement (CMOD) and the von Mises stress field at the maximum CMOD are presented for different cases. The single macroscopic model shows similar results for both mesh sizes (coarse and fine); however, these overestimate by about 18% the maximum load from the multiscale modeling. On the other hand, it can be also observed that the stress concentration changes, being narrower the plastic band in the case of the multiscale model. Regarding the elastic zone, it can be observed that both models are equivalent.

## 4. Proper Generalized Decomposition Multiscale Approaches

The origin of the proper generalized decomposition (PGD) can be found in the large time increment (LATIN) method [131] which was mainly used for solving time-dependent non-linear problems in computational mechanics. The LATIN method was based on the so-called radial decomposition by which the solution field u was sought as a composition of separated variables in space (x) and time (t), as

(64) $$u\left(x,t\right)\approx \overset{N}{\underset{i=1}{\sum }}X_{i}\left(x\right)T_{i}\left(t\right)$$

where N is the number of couples used for the approximation of the solution, and $X_{i}$ and $T_{i}$ are the basis for the space and time functions, respectively.

Later works [132,133,134] proposed a generalization of the decomposition in Equation (64) to any other dimension of the problem, making this approach suitable for multiparametric models. Thus, the separate representation is constructed by means of a successive enrichment of the solution in terms of a N-th summation of the product of D functions (in the case of a D-dimensional problem)

(65) $$u\left(z\right)\approx \overset{N}{\underset{i=1}{\sum }}\overset{D}{\underset{j=1}{\prod }}F_{i}^{j}\left(z_{j}\right)$$

where $F_{i}^{j}$ is the i-th one-dimensional function of the j-th variable $z_{j}$.

As pointed out in [135], the function $F_{i}^{j}\left(z_{j}\right)$ can be defined in terms of a piecewise linear interpolation concerning a total of *n* nodes, and yielding a more general expression for Equation (64). Therefore, for a *D*-dimensional problem with n nodes and a solution approximation of N terms, the total number of unknown becomes NDn, circumventing the curse of dimensionality of classical mesh-based techniques that require $n^{D}$ degrees of freedom.

Recent works include the use of PGD/LATIN model reduction techniques in the analysis of viscoplastic composites [136], cyclic damage [137], and reinforced concrete structures [138] to cite a few.

The PGD formulation is usually defined as a priori since it does not require any knowledge of the solution, it rather works within an iterative strategy as a pseudo-eigenvalue problem [139]. The reader is referred to [140] for a detailed description on the general formulation of this method.

In the work by Ammar et al. [141], the PGD was first used to overcome problems in computational mechanics with different time scales that cannot be separated. The underlying idea is to search for the solution by considering two temporal variables: the first one, t for the coarse scale, and the second one, τ for the fine scale. Therefore, the solution is expressed as

(66) $$u\left(t,\tau \right)\approx \overset{N}{\underset{i=1}{\sum }}F_{i}^{1}\left(t\right)\cdot F_{i}^{2}\left(\tau \right)$$

Since the enrichment at the coarse scale is multiplicative, the continuity between time scales is imposed explicitly. In the paper both penalty functions and Lagrange multipliers are successfully used.

In the authors’ opinion, this technique must be remarked since it is beginning to play an important role in multiscale modeling. Following the structure of this review, PGD is analyzed from the point of view of HM and NHM methods.

### 4.1. PGD in HM Methods

El Halabi et al. [135] presented a homogenization-based multiscale model in which a generic RVE is solved at the microscale by means of the PGD. In such way, the displacement field at the microscale is computed off-line, reducing the computational cost at the macroscale.

The displacement field is defined as the macroscopic field, M, enriched by a microscopic field, m

(67) $$u=u^{M}+u^{m}$$

and consider the spatial coordinates to follow

(68) $$x=x_{m}^{M}+x^{m}$$

where $x^{m}$ denotes the relative position of x with respect to the initial node of the RVE, $x_{m}^{M}$.

Regarding the scales’ information exchange, the micro to macro enrichment is based on the resolution of the RVE at the microscale by means of the PGD, as mentioned before. Thus, the displacement field over each microscopic element is obtained a priori and refers to a macroscopic element. Thus, the boundary conditions of the microstructural RVE and the location of the microscopic RVE with respect to the macroscopic mesh are used as independent variables ($x^{m}$, $x_{m}^{M}$, $u_{m}^{M}$, $u_{m+1}^{M}$).

Then the multiscale problem is reduced to minimizing the residual of the macroscopic nodal forces and internal forces, $p_{ext}\approx p_{int}$. Thus, the internal forces have to be computed

(69) $$p_{int}^{M_{el}}=\overset{N^{m}}{\underset{n=1}{\sum }}\int_{\Omega_{n}^{m}}{\left(B^{M}\right)}^{T}E\frac{\partial u}{\partial x^{m}}dx^{m}$$

**Figure 24:**
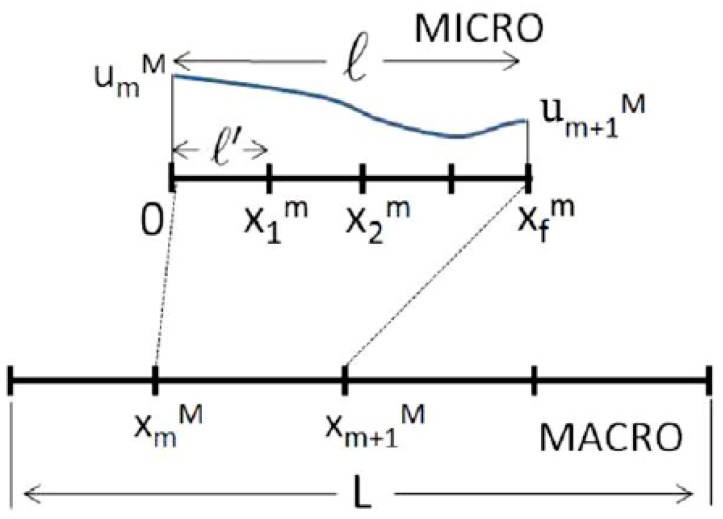
Microscopic and macroscopic discretization in the 1D problem within the proper generalized decomposition. The macro element sizes from $X_{m}^{M}$ to $X_{m+1}^{M}$ and the micro element has its internal resolution with $x_{0}^{m}=x_{m}^{M}$ and $x_{f}^{m}=x_{m+1}^{M}$. Figure taken with permission from [135].

Particularized for the 1D case (see Figure 24); where $B^{M}$ is the strain matrix operator associated with the shape functions in the macroscale, E is the elastic modulus, $N^{m}$ is the total number of elements in the microscale domain, and u can be substituted by the series decomposition.

**Figure 25:**
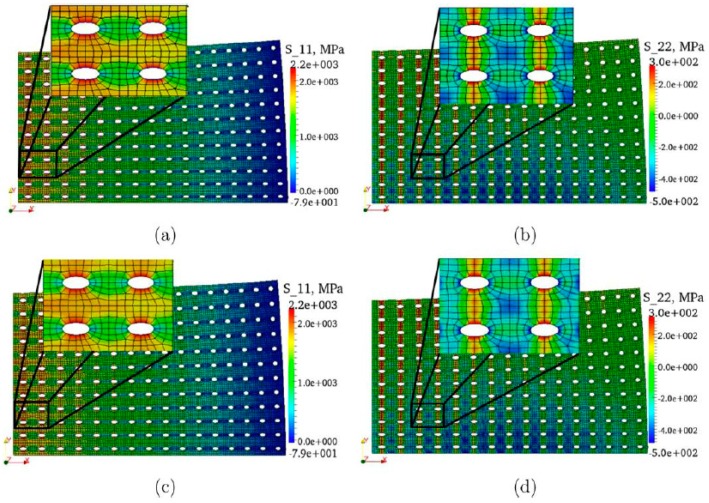
Micro and macroscopic stress distributions for a voided matrix in 2D by means of the proper generalized decomposition. Figure taken with permission from [135].

The homogenization-based multiscale approach proposed by El Halabi et al. [135] is presented for 2D as well (see Figure 25). From the numerical tests, important computer time reduction was obtained. However, the gap to the 3D modeling is somehow larger since the number of independent parameters increases. In any case, the procedure can be extended to dynamic or non-linear problems by including the time or internal variables as extra parameters in the microscale RVE problem. This work has been further extended to account for material nonlinearities in [142].

### 4.2. PGD in NHM Methods

**Figure 26:**
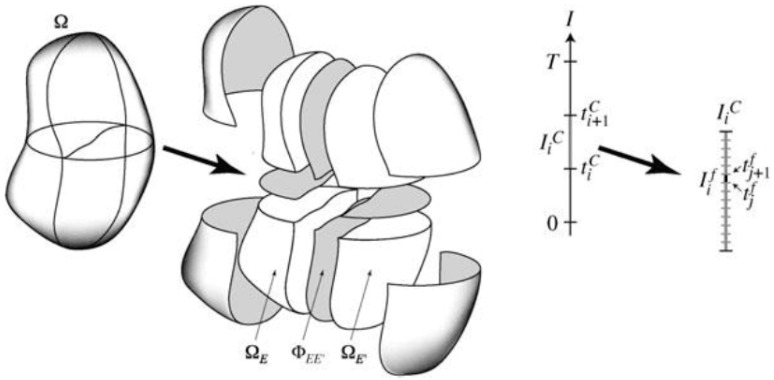
Substructuring of a given domain into other subdomains including interfaces and time discretization. Figure taken with permission from [132].

Among the NHM methods described in Section 3, PGD is especially suitable within a domain decomposition framework. Indeed, in the work by Néron and Ladevèze [132], the idea of using the domain decomposition in order to decompose the structure into an assembly of substructures is already presented (Figure 26), focusing on reducing the computational cost of the PGD approach.

Thus, the macroscale (i.e., bulk) problem is defined in the macroscopic domain, M; while the microscopic problem, m, is modeled by means of PGD, and, in the case of time-dependent problem, must satisfy the following interface conditions:

- Dirichlet: ${\dot{u}}^{m}={\dot{u}}^{M}$ on $\Gamma^{m↔M}$
- Neumann: $\sigma^{m}\cdot n^{m}+\sigma^{M}\cdot n^{M}=0$ on $\Gamma^{m↔M}$

The choice of the parameters at the microscale problem is similar to that in the HM case. Since the displacement field in the microscopic domain is obtained a priori, once the RVE problem is solved, the method performs better than the pure approach.

Finally, regarding the multigrid approach, the use of PGD becomes less competitive since this method is most efficient when fixed meshes are taken into account.

## 5. Multiscale Multiphysical Software

The development of new multiscale multiphysical solving schemes has benefited from the challenges posed by the industry. In fact, this framework has been successfully proven in the automotive [143], aerospace [144], or energy [145] sectors. This has resulted not only in improved numerical strategies, but also in the upgrading of extended-use software (both commercial and open-source) and, even more, the birth of new special purpose tools to carry out advanced simulations.

Numerical modeling is claimed to be as much art as science. For this particular reason, there exist as many tools as practitioners. This aphorism might become more evident when multiscale modeling is taken into account, as it requires the involvement of multidisciplinary teams. Therefore, there does not exist a single (or set of) specific tools to carry out multiscale multiphysical simulations. Yet, the authors propose herein an overview of widely extended software with proven capabilities for this duty.

As described in Section 2, homogenization-based methods rely mainly on the concept of the existence of a representative volume element. For this reason, several software related to the generation of computational microstructures have aroused, for instance, for composites (e.g., Digimat) or polycrystals (e.g., Voro++, Dream3D). In any case, RVEs can be also built within generic finite element preprocessors.

The characterization of RVEs, either within a sequential scheme or an FE^2^ approach, is typically carried out with FE software—such as Ansys [146], Abaqus [147], Comsol [148], etc.—to cite some examples. The interaction between the micro- and macroscale problem can take place within the same simulation environment, or with the use of an external orchestrator. In this sense, the adoption of Python [149] as a scripting language has supposed an important push. Other open-source software that can similarly be used are Kratos Multiphysics [150], Fenics [151], Calculix [152], FEAP [153], or Code Aster [154].

From the perspective of non-homogenization-based methods, the modeling challenge lies on efficient meshing techniques or the enforcement of interface constraints, for instance. 

In the one hand, commercial software such as Ansys, Abaqus, or MSC Patran [155] have typically presented advanced meshing capabilities. If required, high-performance meshing tools such as GiD [156] or Rhino [157], can be used as well. With regard to open-source alternatives, Gmsh [158] is widely extended. On the other hand, built-in capabilities in FE software (e.g., kinematic constraints) can be used to enforce compatibility at the interface of the domains.

Finally, the multiscale solving process is typically time-consuming. For this reason, parallelization techniques (e.g., MPI, CUDA) are a demand in the case of large systems. Moreover, alternative techniques such as that based on the fast Fourier transform (FFT) or the aforementioned PGD are called to play an important role in the upcoming years.

## 6. Conclusions

In this paper, a review of multiscale solvers for continuum problems is presented. Several multiscale strategies of solution, based or not on the homogenization technique, have been visited for a number of problems formulated in continua. The potential interest of each multiscale problem in industry has been emphasized along the paper.

Regarding HM methods these are feasible when length scales separation is assured. Moreover, the solution scheme is different whether the problem is linear or not. In linear problems, the complexity of the method is reduced to compute the macroscopic or apparent properties. However, a nonlinear scheme induces a FE^2^ strategy. That is, each macroscopic Gauss point of the FE mesh calls an FE microscopic simulation on the RVE. Then, the CPU time is dramatically increased under this scenario. In order to amend or minimize this issue, several alternative techniques are under investigation being the PGD one of the most promising ones, which may allow in the near future to run nonlinear multiscale models using conventional computers.

On the other hand, in non-separated scales the so-called NHM methods are invoked. In this paper, several techniques in this field were reviewed, paying special attention to multigrid and domain decomposition within mechanical, heat transfer, or diffusion problems. These methods have been proven efficiently in the literature and the main ingredients of the numerical approach have been discussed.

Due to recent advances and large investigation in multiscale algorithms, this field is becoming applicable in industry. Multiscale simulation is especially well-suited for the design of new concept materials from the microstructure, to get materials with desired or with unprecedented properties, as well as to understand the performance, organization and behavior of hierarchically materials, such as complex multilayered nanocomposites or living tissues, to cite a few.

## Figures and Tables

**Table 1:** Theoretical estimates versus numerical values of effective elastic moduli of a body with spherical voids.

Void Fraction	$C_{1111}^{M}/C_{1111}^{m}$ (Theoretical)	$C_{1111}^{M}/C_{1111}^{m}$ (Numerical)	$C_{1122}^{M}/C_{1122}^{m}$ (Theoretical)	$C_{1122}^{M}/C_{1122}^{m}$ (Numerical)
0.05	0.894	0.897	0.865	0.868
0.1	0.802	0.805	0.748	0.750
0.15	0.722	0.725	0.646	0.648
0.2	0.650	0.652	0.557	0.557
0.25	0.586	0.589	0.479	0.480
0.3	0.527	0.525	0.412	0.406
0.4	0.423	0.413	0.302	0.285
0.5	0.331	0.305	0.219	0.179
